# Insight into Spodium–π Bonding Characteristics of the MX_2_⋯π (M = Zn, Cd and Hg; X = Cl, Br and I) Complexes—A Theoretical Study

**DOI:** 10.3390/molecules27092885

**Published:** 2022-04-30

**Authors:** Meng Gao, Qibo Zhao, Hao Yu, Min Fu, Qingzhong Li

**Affiliations:** 1 College of Chemical and Biological Engineering, Shandong University of Science and Technology, Qingdao 266590, China; z1121365098@126.com (Q.Z.); yuhao3754@hotmail.com (H.Y.); fumin@sdust.edu.cn (M.F.); 2 The Laboratory of Theoretical and Computational Chemistry, School of Chemistry and Chemical Engineering, Yantai University, Yantai 264005, China

**Keywords:** spodium–π bonding, substituent effect, NCI, AIM, electron density shift

## Abstract

The spodium–π bonding between MX_2_ (M = Zn, Cd, and Hg; X = Cl, Br, and I) acting as a Lewis acid, and C_2_H_2_/C_2_H_4_ acting as a Lewis base was studied by ab initio calculations. Two types of structures of cross (**T**) and parallel (**P**) forms are obtained. For the **T** form, the X–M–X axis adopts a cross configuration with the molecular axis of C≡C or C=C, but both of them are parallel in the **P** form. NCI, AIM, and electron density shifts analyses further, indicating that the spodium–π bonding exists in the binary complexes. Spodium–π bonding exhibits a partially covalent nature characterized with a negative energy density and large interaction energy. With the increase of electronegativity of the substituents on the Lewis acid or its decrease in the Lewis base, the interaction energies increase and vice versa. The spodium–π interaction is dominated by electrostatic interaction in most complexes, whereas dispersion and electrostatic energies are responsible for the stability of the MX_2_⋯C_2_F_2_ complexes. The spodium–π bonding further complements the concept of the spodium bond and provides a wider range of research on the adjustment of the strength of spodium bond.

## 1. Introduction

Spodium bond (SpB) [1] refers to the net attractive interaction between a Group 12 element and an electron-rich atom. SpB plays a crucial role in supramolecular chemistry [2,3,4,5,6,7] and crystal engineering [8,9]. For example, a 2D supramolecular polymeric of [Hg_2_L(N_3_)_4_]_n_ prepared with Hg(CH_3_COO)_2_, NaN_3_, and 1,2-bis(pyridin-2-ylmethylene)hydrazine, non-covalent interactions, including spodium bonding, have a defining structure-guiding role. Two new dinuclear Zn(II) complexes [5] were synthesized and characterized. By using a combination of Bader’s quantum theory of ‘‘atoms in molecules’’ (QTAIM) and noncovalent interaction (NCI) method analyses, intramolecular SpBs were characterized and differentiated from coordination bonds [5]. What is more, there is already evidence on the existence and relevance of SpB in biological systems [10,11]. The evidence of spodium bonds in tetrahedral Zn-binding sites [10] demonstrates that Zn SpB’s are abundant and might be involved in protein structures and enzyme inhibition. The strength of three protein models’ SpB interactions in biologically relevant metalloenzymes [11] was estimated by using a combination of QTAIM and NCI plot index computational tools.

After the concept of SpB was proposed, the related experimental and theoretical studies were reported rapidly [12,13,14,15,16,17,18,19,20,21,22]. The non-covalent SpB between the tetracoordinated molecules MX_2_L_2_ (M = Zn, Cd, Hg; X = Cl, Br, I; L = thiourea) and the electron-donating molecules is different from the coordination bonds (anti-bonding Sp–ligand orbital involved). The HgCl_2_⋯L dimers (where L = ClR, SR_2_, PR_3_ families) were explored to unveil the nature of the linear coordinated spodium bonds [12]. In addition, the ability of the HgX_2_ (X = Cl, Br, I) dimer to establish secondary Hg⋯X contacts (spodium bond) and Hg⋯Hg was described [9,13]. The structures, intermolecular interactions, and the spectral variations in the mixtures containing a wide range of compositions (1:3 to 1:14 in molar ratios) of ethanol and ZnCl_2_ was explored to shed light on understanding the properties of the deep eutectic solvents formed by them [14]. What is more, the bi-coordinated molecules ZnX_2_ interact with either a carbene [15,16] or carbodiphosphorane [15] carbon atom, resulting in a C⋯Zn bond. Compared with the beryllium bond, the C⋯Zn bond is not much different than the beryllium bond, and both have a significant covalent contribution [15]. The planar MCl_3_^−^ (M = Zn, Cd, Hg) anions containing a negative π-hole region form a complex with the CN^−^ anion [17] or one another MX_3_^−^ [18,19]. The anion⋯anion spodium bonds and crystal packing forces attribute to some crystal structures [18]. In addition, the SpB coexists simultaneously with other non-covalent interactions in the same system. For example, Liu et al. explored the cooperativity between SpB and pnicogen [20]/chalcogen [21]/tetrel bonds [22] in ternary complexes, and these interactions are strengthened by each other. Accordingly, the electron donors are lone pair electrons and carbenes.

Usually, the π system (ethyne and ethene) is one the candidate electron donor and participates in the σ/π–hole interactions [23,24,25], such as the aerogen bond [24], hydrogen bond [26,27,28,29,30], lithium bond [31,32,33], beryllium bond [34], sodium bond [35], magnesium bond [36], regium bond [37,38,39,40], triel bond [41], tetrel bond [42,43], pnictogen bond [44], chalcogen bond [45], halogen bond [46], and so on [47,48,49]. Wang et al. [50] provided some experimental evidence for the zinc−diborene π interactions in complexes of Zn(II)/Cd(II) with diborene. However, there is no theoretical research on the spodium bonds involved in π systems.

Hence, in this paper, we first study the interaction between MX_2_ (M=Zn, Cd, and Hg; X = Cl, Br, and I) and C_2_H_2_/C_2_H_4_ to explore the dependence of spodium bonding strength on the nature of the M atom and the hybridization of π molecular as well as to unveil the origin of a spodium bond. MX_2_ is a well-known linear molecule which has a large positive electrostatic potential generated on the waist of the Group 12 atom. C_2_H_2_ and C_2_H_4_ have a negative electrostatic potential region above and below the C=C/C≡C. Thus, there should be a strong attraction interaction between them. Considering the stronger electron-withdrawing ability of the F atom and the stronger electron-donating ability of the Li atom, the H atoms in C_2_H_2_ are replaced with –F and –Li, and the corresponding systems were used to study substitution effects on the interactions. What is more, we utilize *sp*-hybridized C_2_H_2_ and *sp*^2^-hybridized C_2_H_4_ interacted with MX_2_ to explore the hybridization effect.

## 2. Theoretical Methods

The second-order Møller–Plesset (MP2) perturbation theory [51] has been a good method for studying intermolecular interactions [24,39,52,53,54,55,56]. Furthermore, the MP2 method was adopted to study spodium bonds [1,18,20,21]. All complexes were optimized using MP2 perturbation theory with aug-cc-pVTZ basis sets [57,58]. Moreover, for iodine, zinc, chromium, and mercury atoms, the aug-cc-pVTZ-PP basis set was adopted to account for relativistic effects. Two types of structures of the cross (**T**) and parallel (**P**) forms were obtained. The frequency analysis at the same computational level was applied to affirm that the optimized geometries corresponded to the ground-state stationary points. It is worth noting that for ethyne and ethane complexes, the global minima corresponded to the **P** form, while the **T** form was a transition state with 1 imaginary frequency. The interaction energy was defined as the difference between the energy of the complex and the energies of the monomers with their geometries taken from the complex. Using the counterpoise method proposed by Boys and Bernardi [59], the interaction energy was corrected for the basis set superposition error (BSSE). All calculations were performed using Gaussian 09 package [60]. The interaction energy was also analyzed at the MP2/aug-cc-pVTZ (PP) level by the localized molecular orbital energy decomposition analysis (LMOEDA) method [61] with GAMESS program [62]. The total interaction energy can be decomposed into 5 components: electrostatic (ES); exchange (EX); repulsion (REP); polarization (POL); and dispersion (DISP) energies.

Molecular electrostatic potentials (MEPs) on the 0.001 electronsbohr^−3^ contour of the electronic density were calculated with the Multiwfn [63] at the MP2/aug-cc-pVTZ(PP) level. To obtain a deeper insight into the interaction nature of these complexes in the light of charge transfer and orbital interactions, we performed natural bond orbital (NBO) [64] analyses included in Gaussian 09. Quantum theory of “atoms in molecules” (QTAIM) analysis [65] was performed with the Multiwfn [63] to obtain topological parameters of the bond critical point (BCP), including electron density (*ρ*), its Laplacian (▽^2^*ρ*), and total energy density (*H*). Since the electron density is at a maximum at the nuclei, the localization of maxima enables the identification of atomic positions. The first-order saddle points between the maxima are usually known as bond critical points (BCPs) [34,65]. The wavefunctions were used to perform topological analyses for these complexes, including non-covalent interaction (NCI). NCI maps were plotted with the VMD program [66]. NCI involves the reduced density gradient (RDG) and the electron density (*ρ*). RDG is defined as:

(1) $$\mathrm{RDG}=\frac{1}{2{\left(3\pi^{2}\right)}^{1/3}}\frac{\left|\nabla \rho \right|}{\rho^{4/3}},$$

## 3. Results and Discussion

### 3.1. Molecular Electrostatic Potential of Monomers

**Figure 1:**
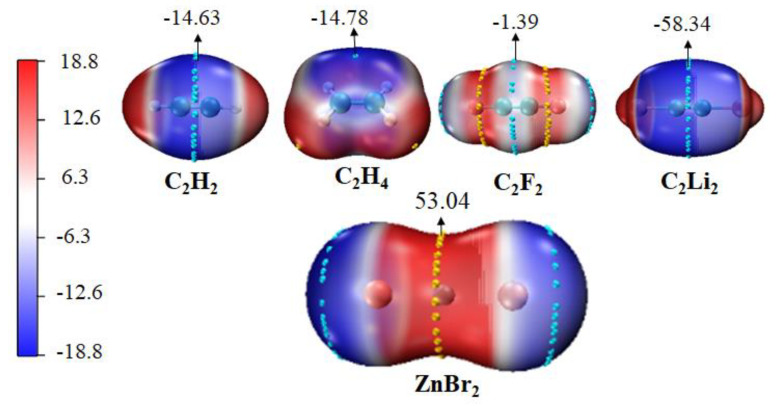
MEP surfaces of ZnBr_2_ and π systems at the MP2/aug-cc-pVTZ(PP) level of theory at the 0.001 electrons Bohr^−3^. Cyan and yellow balls on the surface correspond to *V*_min_ and *V*_max_, respectively. All are in kcal/mol.

Molecular electrostatic potential (MEP) is a very effective method for predicting the possibility of intermolecular interactions, which could ascertain the most appropriate interaction region of each monomer [12,20,67,68,69]. Figure 1 depicts the MEP maps of ZnBr_2_ and π systems. It is evident that the most positive MEP (*V*_max_) was generated on the waist of Zn atom in ZnBr_2_, which was similar to the rest of the MX_2_ monomers (M = Zn, Cd, Hg; X=Cl, Br, I), and the most negative MEP (*V*_min_) was between two carbons in the π systems. There should be a strong attraction between the M atom and a π system. The values of *V*_max_ and *V*_min_ for monomers are collected in Table 1. It is immediate that for the MX_2_ with the same M and different halogen atoms, the value of *V*_max_ on the M atom increased in the order of I < Br < Cl; and for the ones with the same halogen atom, *V*_max_ became more positive in the order of Hg < Zn < Cd, likely due to the 4d series moderate polarizability and primogenic repulsion from inner core d-electrons [70,71]. This indicates that the value of *V*_max_ on M atom became more positive as the halogen was less polarizable. The *V*_min_ of *sp*^2^-hybridized ethylene was slightly more negative than that in acetylene. For the acetylene system, electron-withdrawing atom F increased the *V*_min_, and electron-donor atom Li became *V*_min_ more negative.

### 3.2. Geometrics and Interaction Energies

**Figure 2:**
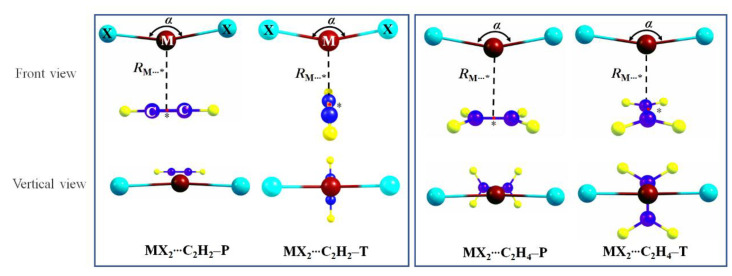
Schemes of spodium–π bonded binary complexes. Dark red is the M atom (M = Zn, Cd, Hg), cyan is halogen atom X (X = Cl, Br, I), blue is C atom and yellow is H atom. Binding distance *R*_M⋯_* between the M in MX_2_ and the centers of C–C bond in π molecules (* denotes the center of C–C bond). The angle of X–M–X in MX_2_ is *α*. Dihedral angle of X–X⋯C–C is marked by *θ*.

The binary complexes between MX_2_ (M = Zn, Cd, and Hg; X = Cl, Br, and I) and acetylene or ethylene may adopt two different forms depending on the relative orientation of the MX_2_ subunit with respect to the unsaturated molecule (Figure 2). It should be noted that the T-form complexes are transition states with an imaginary frequency, while P-form complexes are global minima. In MX_2_⋯C_2_H_2_–T or MX_2_⋯C_2_H_4_–T (**T** form), the X–M–X axis adopts a cross configuration (*θ* is 82°–83°) with the molecular axis of C≡C or C=C but is parallel (*θ* = 0°) with the latter in MX_2_⋯C_2_H_2_–P and MX_2_⋯C_2_H_4_–P (**P** form). These forms are similar to previous work of π–beryllium bonds [34], π–magnesium bonds [36], and aerogen–π bonds [24]. The geometric parameters of these binary complexes and corresponding monomers are shown schematically in Figure 2 and in Figure S1, and the corresponding values are listed in Table 2 and Table S1, together with the interaction energy.

In all complexes, the MX_2_ subunit, which is linear for the isolated molecule, becomes nonlinear, with X–M–X angle *α* between 143° and 176°. We attribute this obvious bending to the charge transfer between the highest π-occupied orbitals of the unsaturated moiety and the lowest unoccupied orbitals of the MX_2_ moiety. The *α* in **P** form is smaller than that in the **T** form. For example, the *α* is 149° in ZnBr_2_⋯C_2_H_2_–P and 158° in ZnBr_2_⋯C_2_H_2_–T. This indicates that there is a stronger charge transfer in **P** form than that in the **T** form. What is more, the curvature increases with the order Hg, Cd, and Zn, and the bending diminishes with the decrease of halogen atomic number.

The binding distance *R*_M⋯*_ defined as the distance between the M atom of MX_2_ and the center of C–C bond in C_2_H_2_ or C_2_H_4_ is in the wide range of 2.4–3.1 Å, which is longer than that in MCO_3_ (M = Zn, Cd, Hg)⋯nitrogen-containing bases (HCN, NHCH_2_, NH_3_) (1.9–2.2 Å) [20] and ZnX_2_⋯carbene (2.0–2.2 Å) [15] or (2.12–2.20 Å) [16]. It shows that there is a spodium–π bond between MX_2_ and C_2_H_2_ or C_2_H_4_. In general, the *R*_M⋯*_ becomes shorter in the order of Hg→Cd→Zn. For example, ZnX_2_⋯C_2_H_2_–P (2.387–2.406 Å) is shorter by 0.3 Å than that in CdX_2_⋯C_2_H_2_–P (2.664–2.700 Å) and 0.7 Å in HgX_2_⋯C_2_H_2_–P (3.056–3.145 Å). In addition, with the increase of the halogen atomic number, the *R*_M⋯_* increases by 0.04 Å in CdX_2_⋯C_2_H_2_–P and 0.09 Å in HgX_2_⋯C_2_H_2_–P, although it decreases by 0.02 Å in ZnX_2_⋯C_2_H_2_–P. Compared with MX_2_⋯C_2_H_2_–P dimers, the *R*_M⋯_* of MX_2_⋯C_2_H_2_–T has an elongation (0.02−0.19 Å) except ZnBr_2_⋯C_2_H_2_–T and ZnI_2_⋯C_2_H_2_–T. The M⋯* distance of MX_2_⋯C_2_H_4_–P shortens by 0.01−0.05 Å than that in MX_2_⋯C_2_H_2_–P. Compared to MX_2_⋯C_2_H_4_–P, the change of *R*_M⋯_* in MX_2_⋯C_2_H_4_–T is about ~0.1 Å.

The interaction energies Δ*E* between MX_2_ and C_2_H_2_ or C_2_H_4_ are larger in the order of Hg < Cd < Zn (from −1.37 kcal/mol to −11.68 kcal/mol at the MP2/aug-cc-pVTZ level), although weaker than the interaction energy of MCO_3_ (M = Zn, Cd, Hg)⋯nitrogen-containing bases (HCN, NHCH_2_, NH_3_) complex (−31 kcal/mol to −56 kcal/mol at the MP2/aug-cc-pVTZ level) [20], and dissociation energy of ZnX_2_⋯carbene (10–79 kcal/mol at the *ω*B97X-D/6-311++G(2df,2p) level) [15] or (18.5–27.4 kcal/mol at the MN15/6-311+G(d) level) [16]. The Δ*E* of the Zn-containing binary is 3–6 times that of the Hg-containing binary. The order of Δ*E* is inconsistent with the order of *V*_max_ value of the electrostatic potential of M (Hg < Zn < Cd). We predict that there are other types of interaction components besides the electrostatic one, which is dominant in the formation of the spodium bond [20]. The coexistence of strong attractive (blue regions), weak attractive (green regions), and strong repulsion (red regions) interactions (Figures S2–S5, in Section 3.5) seems to prove this. In addition, the interaction energies are stronger in the order of I < Br < Cl. This trend of interaction energies could be demonstrated by the electrostatic interactions between the positive MEPs of Zn/Cd/Hg and the negative ones of C_2_H_2_ or C_2_H_4_. The interaction energy analysis shows that both hybridizations have a small difference as well as. The *V*_min_ of C_2_H_2_ (−14.63 kcal/mol) and C_2_H_4_ (−14.78 kcal/mol) is similar, which explains the aforementioned phenomenon. It indicates that the carbon hybridization of the Lewis base has a faint effect on the spodium–π bond.

### 3.3. Substituent Effect

To explore the substitution effect on the spodium–π bond, we replaced the H atom in MX_2_⋯C_2_H_2_–P with F or Li. The interactions in the F-substituted complexes MX_2_⋯C_2_F_2_–P became weaker than that in MX_2_⋯C_2_H_2_–P, characterized by a longer binding distance *R*_M⋯_* (~0.5 Å in ZnX_2_⋯C_2_F_2_–P, ~0.3 Å in CdX_2_⋯C_2_F_2_–P, less than 0.1 Å in HgX_2_⋯C_2_F_2_–P) and smaller interaction energy (−0.79 kcal/mol to −3.25 kcal/mol: in the order of Hg < Zn < Cd). This was due to the large electron-withdrawing ability of F, which significantly decreased the negative MEPs of C_2_F_2_. As a consequence, the interaction energies between MX_2_ and C_2_F_2_ became weaker, with a long binding distance. The order of interaction energy was consistent with the order of *V*_max_ value on M (Hg < Zn < Cd), and the decreased magnitude of interaction energy in MX_2_⋯C_2_F_2_–P was in the order of Zn (~8 kcal/mol) < Cd (~6 kcal/mol) < Hg (~1 kcal/mol). This confirms the important role of electrostatic interactions in MX_2_⋯C_2_F_2_–P system. In addition, the *α* close to 180°, and the dihedral angle *θ* increased from 0° to 33–52°. It indicates that the repulsion interactions decreased. This is illustrated by the original red regions turning orange in Figure S6.

On the contrary, the interaction for MX_2_⋯C_2_Li_2_–P was strengthened with a shorter binding distance and larger interaction energy (approximately 9–20 times). The change of binding distance *R*_M⋯_* in MX_2_⋯C_2_Li_2_–P was obvious, shortened by 0.4 Å in ZnX_2_⋯C_2_Li_2_–P, 0.5 Å in CdX_2_⋯C_2_Li_2_–P, 0.9 Å in HgX_2_⋯C_2_Li_2_–P. The shorter binding distance was ascribed to the increase of electrostatic interaction between MX_2_ and C_2_Li_2_, resulting in the more negative MEPs of C_2_Li_2_. Compared with MX_2_⋯C_2_H_2_–P, although the increased multiple of interaction energy was ~9 times for ZnX_2_⋯C_2_Li_2_–P, ~10 times for CdX_2_⋯C_2_Li_2_–P, and ~25 times for HgX_2_⋯C_2_Li_2_–P, the interaction energies for MX_2_⋯C_2_Li_2_–P were stronger in the order of Hg < Cd < Zn. What is more, the molecular deformation for MX_2_⋯C_2_Li_2_–P (α: 117–134°) was more significant than that in MX_2_⋯C_2_H_2_–P, which can be chalked up to strong interaction energies. It shows that other forms of interaction components (Figure S7) beyond electrostatic effects also play an important role.

The charge transfer CT in the complexes is presented in Table S2. The negative charge transfer confirms the Lewis acid roles for the MX_2_ molecules. The charge transfer in ZnX_2_⋯C_2_H_2_–P and CdX_2_⋯C_2_H_2_–P is about −0.06 e and is about −0.02 e for HgX_2_⋯C_2_H_2_–P. F substituents decrease the charge transfer (−0.020 e to −0.043 e) due to their electron-withdrawing nature, whereas the electron donor Li increases the charge transfer (−0.870 e to −1.293 e).

The second-order perturbation energies (*E*^(2)^) of spodium–π interaction is analyzed with mainly orbital interaction BD_C≡C_→LP*_M_, where BD_C≡C_ denotes the C≡C bonding orbital, and LP^*^_M_ is the lone pair anti-bonding orbital of the Group 12 atom. Accompanied with these orbital interactions, there occurs a charge transfer from ethyne/ethene to MX_2_, confirmed by the negative charge on MX_2_ [15,24]. The Group 12 elements are usually considered to be post-transition or main group elements and are not labeled as transition metals [72]. Using the traditional DCD model [73] cannot interpret well the spodium–π interaction. It is necessary to point out that there are other orbital interactions in the spodium–π interaction. The orbital interactions in HgX_2_⋯C_2_H_2_–P are much weaker than those in CdX_2_⋯C_2_H_2_–P and ZnX_2_⋯C_2_H_2_–P, indicating the partial covalent characteristics of the latter two systems. For the same metal in MX_2_, *E*^(2)^ (BD_C≡C_→LP*_M_) becomes higher with the increase of X atomic number, which is in the reverse order of the corresponding systems’ interaction energy. We attribute this inconsistency to the coexistence of spodium bond, the X∙∙∙H interactions, and coulomb repulsive interaction.

### 3.4. AIM

The existence of spodium–π interaction is further characterized with the presence of BCPs between the M atoms and π system (Figure S8). Electron density (*ρ*), Laplacian (▽^2^*ρ*), and total energy density (*H*) at the intermolecular BCPs of the complexes are listed in Table 3 and Table S3. The *ρ* value for the spodium–π interaction containing Zn and Cd is about 0.03−0.04 au, which is smaller than that in MCO_3_ (M = Zn, Cd, Hg)⋯nitrogen-containing bases (HCN, NHCH_2_, NH_3_) complex (0.08 au) [20], and of ZnX_2_ (X = H, Me, Et or F, Cl, Br) with carbenes or carbodiphosphoranes (0.07−0.11 au) [15,16]. For most complexes, the electron density is larger with the increase of the interaction energy. However, this law is not applicable to the Zn containing systems, mainly because of the existence of strong repulsion resulting from the orbital interaction between the Zn–X bonding and C–H bonding of C_2_H_2_/C_2_H_4_. Hence, the strength of spodium–π bond in most cases can be measured by the topological parameters, particularly the electron density. F substituents prominently decrease the electron density of Zn and Cd systems due to their electron-withdrawing nature, whereas the electron density changes slightly for the system containing Hg. Compared with MX_2_⋯C_2_H_2_–P, the stronger electron donor Li increases the electron density by approximately 2–6 times, reached up to 0.07−0.08 au. The values of *ρ* increase in the order of Hg < Cd < Zn, which is the same as the order of interaction energies.

It has been confirmed that the type of interactions can be classified in the light of the sign of ▽^2^*ρ* and *H* [74]. Positive ▽^2^*ρ* and *H* indicate a purely closed-shell interaction. The partially covalent nature can be confirmed by the positive ▽^2^*ρ* and negative *H* [75,76]. For the spodium–π interaction of HgX_2_⋯C_2_H_2_–P, HgX_2_⋯C_2_H_2_–T, and HgX_2_⋯C_2_F_2_–P, HgX_2_⋯C_2_H_4_–P in which Hg is the Lewis acid, both ▽^2^*ρ* and *H* are positive, corresponding to a purely closed-shell interaction. However, the ▽^2^*ρ* is positive and the *H* is negative for the complexes containing Zn and Cd, indicating a partially covalent interaction [74,77,78]. The SpB involving lone pairs [20], anions [18], carbenes, or carbodiphosphoranes [15] are also characterized by a degree of covalence. Thus, in combination with the high interaction energies as discussed above, spodium–π interaction containing Zn and Cd has a partially covalent nature. Both ▽^2^*ρ* and *H* are positive in MX_2_⋯C_2_F_2_–P, corresponding to a purely closed-shell interaction. Positive ▽^2^*ρ* and negative *H* confirm the partially covalent nature interaction for MX_2_⋯C_2_Li_2_–P. This shows that the substituent atoms of π system change the nature of spodium–π interaction.

**Figure 3:**
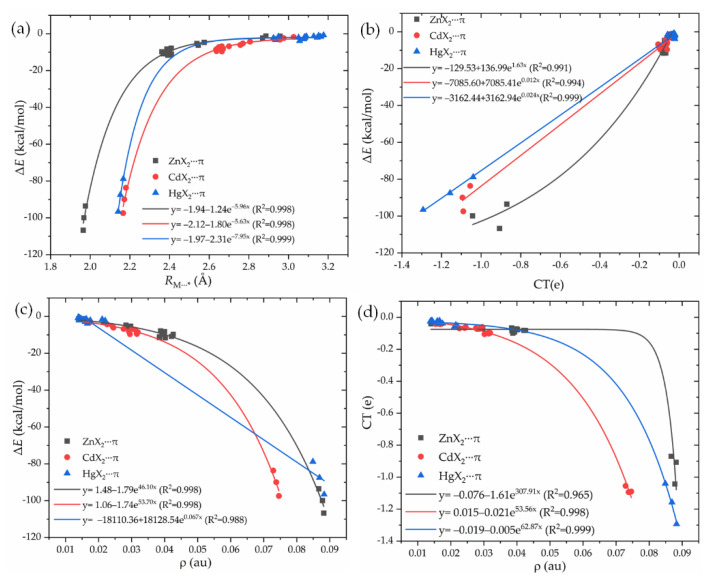
The relationships between the interaction energy Δ*E* and the binding distance *R*_M⋯_* (**a**)_,_ charge transfer CT (**b**), or the electron density ρ at the BCP (**c**), and the relationships between CT and ρ at the bond critical point (**d**).

It is interesting to check the quality of the exponential relationship between the interaction energy Δ*E* and the binding distance *R*_M⋯_*, or the charge transfer CT, or the electron density *ρ* at the BCP. Three of all (Figure 3a–c) display an exponential relationship with the Δ*E*, with a correlation coefficient of ~0.99. Thus, they can be used to estimate the change of spodium–π interaction strength. In addition, there is an exponential relationship between CT and *ρ* (Figure 3d). A greater value of *ρ* means more charge transfer between MX_2_ and π systems.

### 3.5. NCI Analyses

**Figure 4:**
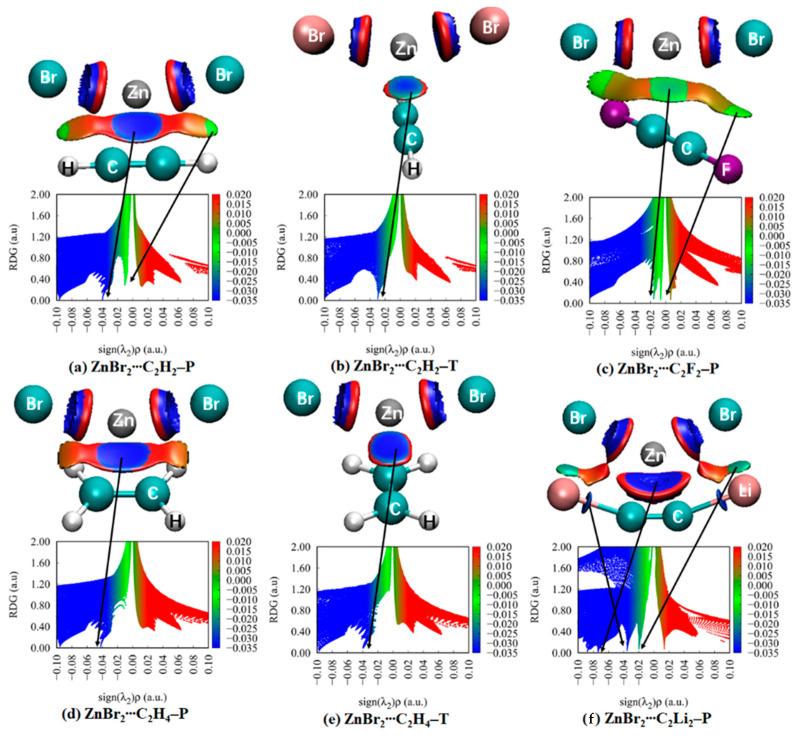
Plots of the reduced density gradient RDG versus the electron density multiplied by the sign of the second Hessian eigenvalue (sign(λ_2_)*ρ*) of the selected binary complexes. NCI maps of the corresponding binary complexes. Blue, green, orange, and red areas correspond to strong attractive, weak attractive, weak repulsive, and strong repulsive interactions, respectively.

Noncovalent interaction analysis (NCI) provides the graphical visualization of the regions where non-covalent interactions occur in real-space, which was capable of distinguishing van der Waals interactions and repulsive steric interactions [79,80]. NCI analysis also offers continuous surfaces through color codes, which is able to recognize the attractive or repulsive nature of the interactions and to decide their relative strength on a qualitative but visual basis. To our knowledge, this technique was carried out for the spodium bond [4,15,16,20]. Thus, we are interested in the deep insights that NCI method can provide for the complexes between MX_2_ and π systems. NCI analysis for some complexes is depicted in Figure 4, and the analysis for all complexes is shown in Figures S2–S7. The color-mapped isosurfaces and corresponding scatter diagrams of RDG versus sign(λ_2_)*ρ* for the investigated complexes are also given.

In MX_2_∙∙∙C_2_H_2_–P (Figure S2), there are two spikes in the negative value of the abscissa. The more negative one (about −0.04 a.u. for Zn in Figure S2a–c, −0.03 a.u. for Cd in Figure S2d–f, −0.02 a.u. for Hg in Figure S2g–i) represents the spodium–π interaction, characterized by a large blue or green disc. The less negative spike (−0.01 a.u.) is referred to the two regions of weak X∙∙∙H interaction between the H of C_2_H_2_ and X of MX_2_, shown by flakes. Evidently, the weak X∙∙∙H interaction cannot be detected in the AIM but can with the NCI method. The former interaction is evidently stronger than that of the latter. Between the two interactions mentioned above, a red or orange region indicates a repulsive interaction, which accounts for the coulomb repulsive interaction. In MX_2_∙∙∙C_2_H_2_–T (Figure S3), there is only one spike in the negative value of the abscissa representing spodium–π interaction. Moreover, the spike in MX_2_∙∙∙C_2_H_2_–T is less negative than that in the P form. It indicates that the spodium–π interaction in the P form is stronger than that in the T form. For MX_2_∙∙∙C_2_H_4_–P (Figure S4), the spodium–π spike (−0.02 a.u. to −0.04 a.u.) and repulsive spike are analogous to MX_2_∙∙∙C_2_H_2_–P. The spike of X∙∙∙H interactions disappears in ZnX_2_∙∙∙C_2_H_4_–P (Figure S4a–c) and CdX_2_∙∙∙C_2_H_4_–P (Figure S4d–e) and appears in HgX_2_∙∙∙C_2_H_4_–P (Figure S4f–g). For MX_2_∙∙∙C_2_H_4_–T (Figure S5), the spodium–π spike and repulsive spike in a narrow region is present, and the X∙∙∙H interactions’ spike is disappeared.

For F-substituted systems, there are similarities with MX_2_∙∙∙C_2_H_2_–P. The MX_2_∙∙∙C_2_F_2_–P complexes (Figure S6) have two attractive and one repulsive spikes. The spike representing the spodium–π interactions moves to the right direction of the X-axis than that in MX_2_∙∙∙C_2_H_2_–P, indicating the weakening of spodium–π interaction. The X∙∙∙F interactions are also shown (less −0.01 a.u.), which is weaker than the X∙∙∙H interactions in MX_2_∙∙∙C_2_H_2_–P. For the Li system (Figure S7), the spodium–π interaction spike moves to the more negative of the X-axis (−0.06 a.u. to −0.08 a.u.) than MX_2_∙∙∙C_2_H_2_–P, indicating the enhancement of the spodium–π interaction. The X∙∙∙Li interaction spikes are on the −0.02 a.u. Two additional blue discs around −0.04 a.u. between Li and C in C_2_Li_2_ indicate attractive interactions.

### 3.6. Electron Density Shift

**Figure 5:**
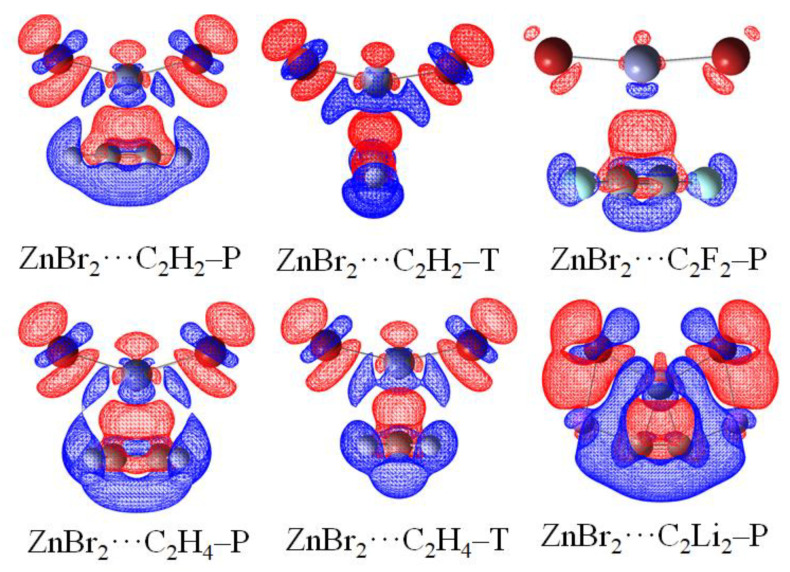
Electron density shifts of selected binary complexes (iso = ± 0.0008). Red regions indicate increased electron density, while blue regions represent decreased electron density.

Figure 5 illustrates the maps of electron density shifts in the ZnBr_2_⋯π systems, which are generated by the difference between the density of the optimized complex and the sum of individual monomers in their same internal geometries. The electron density shifts of all complexes are shown in Figures S9–S14. The increases in density arising from the interaction are illustrated in the red regions and losses shown in the blue areas. The most prominent feature is a red region in the interaction space M⋯π and a blue region of density depletion on the M atom. This feature indicates the presence of spodium–π interaction. It is found that there is a buildup of the electron density on the halogen atoms in MX_2_ and electron loss occurs on the H/F/Li atoms in π systems. This confirms the X⋯H/F/Li interaction. Besides, density depletion is observed on the M–X of MX_2_. Considering the electron loss on the H/Li atoms in π systems, it provides evidence for the steric interactions between the H/Li atoms and the M–X.

### 3.7. Energy Decomposition

A decomposition of interaction energy provides valuable insight into understanding the physical pictures of all binary complexes. The physical components from GAMESS, including electrostatic (ES), exchange (EX), repulsion (REP), polarization (POL), and dispersion (DISP) energies for some representative complexes, are presented in Table 4. More details are seen in Table S4 and Figure S15. Between MX_2_ and C_2_H_2_/C_2_H_4_, the remarkable overlap of molecular orbitals results in a large EX and a much larger REP. The exchange energy is chiefly caused by the overlap of molecular orbitals, and it is more negative in the **P** form than in the **T** form. Considering both EX and REP are mutually dependent, the following discussion is not focused on them. For most complexes (Figure S15a–d), the magnitude of ES is more negative than POL and DISP, indicating that the electrostatic interaction is dominant to the total interaction energy of the spodium–π bonding. For Zn/Cd containing complexes, three attractive terms (ES, POL, and DISP) become more negative in the order of DISP < POL < ES, and ES is about twice as much as POL. However, for the Hg complexes, the order is POL < DISP < ES, and POL is nearly equal to DISP. It indicates that the contribution of POL and DISP cannot be ignored. On the other hand, with the variation of halogen substituent for a given Group 12 metal, a little energy difference is found in the energy components, that is, halogen substituents have little effect on the nature of the spodium–π interaction.

The strong electron-withdrawing group F (Figure S15e) causes a great reduction of ES and POL, especially in ZnX_2_⋯C_2_F_2_–P and CdX_2_⋯C_2_F_2_–P. In both systems, ES is the most negative, followed by DISP, and POL is the least negative. The ES contribution is comparable with that of DISP. However, in HgX_2_⋯C_2_F_2_–P, the order is DISP > ES > POL, which is inconsistent with that in HgX_2_⋯C_2_H_2_–P, indicating that the DISP contribution is dominant. Interestingly, Li group results in a sharp increase of ES and POL and has a slight effect on DISP. Moreover, the MX_2_⋯C_2_Li_2_–P complexes are dominated by electrostatic energy. The relatively large POL (–80.70 kcal/mol to −119.31 kcal/mol) suggests that the orbitals undergo a significant change in their shapes, a typical character in the formation of covalent bonds, conforming the partially covalent nature of spodium–π interaction in MX_2_⋯C_2_Li_2_–P (Figure S15f). Obviously, the substituent of Lewis acid has a prominent effect on the nature of spodium–π interaction.

### 3.8. Comparison

There are a number of reports about the interactions including π systems, such as π–hydrogen bond [26,27,28,29,30], π–lithium bond [31,32,33], π–beryllium bond [34], π–triel bond [41], π–tetrel bond [42,43], regium–π bond [37,38,39,40], π–pnictogen bond [44], π–chalcogen bond [45], π–halogen bond [46], aerogen–π bond [24], π–sodium bond [35], π–magnesium bond [36], and so on [47,48,49]. We compare the binding distance and interaction energy of some complexes. In Table 5, the shortest binding distance is ~0.93 Å in π–hydrogen bond, and the longest binding distance is ~3.3 Å in π–tetrel bond. Although the binding distances of both are different, the interaction energies are relatively weak at −2.5 kcal/mol to −2.8 kcal/mol. When different Lewis acids interact with acetylene/ethylene, the strength of interaction can be adjusted. For example, the interaction energy reaches to −11.68 kcal/mol in Cl_2_Zn⋯C_2_H_4_–P (MP2/aug-cc-pVTZ) and even to −58.79 kcal/mol in FAu⋯C_2_H_4_ (CCSD(T)/aug-cc-pVTZ//*ω*B97XD/aug-cc-pVTZ). The value of Δ*E*_regium–π_ is about five times of Δ*E*_spodium–π_, and Δ*E*_spodium–π_ is about five multiples of Δ*E*_π–hydrogen_ at the same calculated level (MP2/aug-cc-pVTZ). It shows that the interaction energies of spodium–π bond are moderate, which is similar to π–magnesium bond.

In addition, we compare the geometric energetic of SpB formed by molecules containing Group 12 atoms and varied molecules, such as lone-pair-containing molecules [1,11,12,14,20], anions [18], carbenes, and carbodiphosphoranes [15], and π systems. Some typical SpB’s examples are listed in Table 6. In these complexes of SpB, the binding distance *R* is in a range of 1.879–3.616 Å and the interaction energy ranges from −0.79 kcal/mol to −106.75 kcal/mol. The longest distance presents in the anionic dimer (HgCl_3_^−^)_2_ in water medium (3.62 Å), and the interaction energy is only −1.88 kcal/mol [18]. This anionic crystal structure attributes to the noncovalent spodium bonds and crystal packing forces. The interaction energy provides a wide range from −2.2 kcal/mol to −56.68 kcal/mol for the SpB involving lone pair electrons [1,20], which is dominated by electrostatics or polarization [20]. The π systems as a Lewis base participate in the formation of SpB, which further complements the concept of spodium bond. The adjustment of the SpB’s strength in a wider range (−0.79 kcal/mol to −106.75 kcal/mol) is realized.

## 4. Conclusions

The MX_2_ as the Lewis acid engages in spodium–π bonds with π systems, and two different binding types (**P** or **T** form) are obtained. These complexes between the MX_2_ (M = Zn, Cd, and Hg; X = Cl, Br and I) and π system were investigated in view of the equilibrium structure, energetics, electrostatic potential, AIM, NCI, and energy decomposition. The total interaction consists of several parts: spodium–π interaction, X⋯H/F/Li weak interaction and repulsion interaction. Spodium–π interaction is the principal, while the other two should not be ignored. Generally, the spodium–π interaction becomes stronger in the order Hg < Cd < Zn. The enhancing effect of the halogen substituent on the MX_2_ is more prominent in the order of I < Br < Cl. With the increase of electronegativity of the substituents on the Lewis acid, the interaction energies increase. For the Lewis base, the interaction energies decrease with increasing electronegativity of the substituents. The nature of interactions is related to the Group 12 metals and π systems. The spodium–π interaction of complexes containing C_2_H_2_ or C_2_H_4_ is dominated by electrostatic energy, especially for Zn and Cd with a partially covalent contribution, while a purely closed-shell nature for Hg. In the complexes containing C_2_F_2_, dispersion is comparable with that of electrostatic. Even in HgX_2_⋯C_2_F_2_–P, dispersion contribution exceeds that of electrostatic energy. It is worth mentioning that no one plays the sole role. Our results further extend the concept of spodium bond and provide support for the adjustment of the spodium bonds’ strength in a wider range.

## Figures and Tables

**Table 1:** The most positive (*V*_max_) and negative (*V*_min_) MEPs on the M atoms and π systems. All in kcal/mol.

Monomers	*V*_max_	Monomers	*V*_min_
ZnCl_2_	59.24	C_2_H_2_	−14.63
ZnBr_2_	53.04	C_2_H_4_	−14.78
ZnI_2_	45.56	C_2_F_2_	−1.39
CdCl_2_	63.29	C_2_Li_2_	−58.34
CdBr_2_	58.61		
CdI_2_	51.82		
HgCl_2_	44.54		
HgBr_2_	41.46		
HgI_2_	37.45		

**Table 2:** Binding distance (*R*_M⋯_* in Å) between the M in MX_2_ and the centers of C–C bond in π molecules (* denotes the center of C–C bond), M−X (*R*_M–X_ in Å) and C−C bond length (*R*_C–C_ in Å) of complexes, X-M-X angle (*α* in degrees), and dihedral angle of X–X⋯C–C (*θ* in degrees), and interaction energies (Δ*E*, kcal/mol) in the complexes.

Complexes	*R*_M⋯_*	*R*_M–X_	*R*_C–C_	*α*	*θ*	Δ*E*
ZnBr_2_⋯C_2_H_2_–P	2.387	2.222	1.219	149	0	−9.72
CdBr_2_⋯C_2_H_2_–P	2.670	2.401	1.218	161	0	−8.26
HgBr_2_⋯C_2_H_2_–P	3.078	2.363	1.215	175	0	−2.96
ZnBr_2_⋯C_2_H_2_–T	2.538	2.204	1.216	158	83	−5.29
CdBr_2_⋯C_2_H_2_–T	2.770	2.389	1.216	165	83	−5.24
HgBr_2_⋯C_2_H_2_–T	3.083	2.359	1.214	176	84	−1.78
ZnBr_2_⋯C_2_H_4_–P	2.367	2.225	1.347	146	0	−10.89
CdBr_2_⋯C_2_H_4_–P	2.635	2.406	1.344	157	0	−8.58
HgBr_2_⋯C_2_H_4_–P	3.051	2.364	1.338	174	0	−2.12
ZnBr_2_⋯C_2_H_4_–T	2.400	2.216	1.346	153	82	−9.51
CdBr_2_⋯C_2_H_4_–T	2.640	2.401	1.344	163	82	−8.17
HgBr_2_⋯C_2_H_4_–T	2.924	2.365	1.339	175	82	−2.30
ZnBr_2_⋯C_2_F_2_–P	2.886	2.184	1.199	171	42	−1.26
CdBr_2_⋯C_2_F_2_–P	2.976	2.376	1.199	174	40	−2.39
HgBr_2_⋯C_2_F_2_–P	3.152	2.355	1.197	179	46	−1.03
ZnBr_2_⋯C_2_Li_2_–P	1.968	2.348	1.276	119	0	−99.94
CdBr_2_⋯C_2_Li_2_–P	2.172	2.534	1.279	132	0	−89.97
HgBr_2_⋯C_2_Li_2_–P	2.152	2.543	1.289	126	0	−87.51

**Table 3:** Electron density (*ρ* in au), Laplacian (▽^2^*ρ* in au), and total energy density (*H* in au) at the intermolecular bond critical points (BCPs) in the complexes.

Complexes	*ρ*	▽^2^*ρ*	*H*
ZnBr_2_⋯C_2_H_2_–P	0.0398	0.1023	−0.0076
CdBr_2_⋯C_2_H_2_–P	0.0292	0.0863	−0.0018
HgBr_2_⋯C_2_H_2_–P	0.0160	0.0480	0.0009
ZnBr_2_⋯C_2_H_2_–T	0.0297	0.0754	−0.0037
CdBr_2_⋯C_2_H_2_–T	0.0242	0.0708	−0.0006
HgBr_2_⋯C_2_H_2_–T	0.0157	0.0468	0.0009
ZnBr_2_⋯C_2_H_4_–P	0.0421	0.0893	−0.0091
CdBr_2_⋯C_2_H_4_–P	0.0319	0.0817	−0.0030
HgBr_2_⋯C_2_H_4_–P	0.0175	0.0463	0.0005
ZnBr_2_⋯C_2_H_4_–T	0.0393	0.0843	−0.0078
CdBr_2_⋯C_2_H_4_–T	0.0315	0.0807	−0.0028
HgBr_2_⋯C_2_H_4_–T	0.0220	0.0570	−0.0003
ZnBr_2_⋯C_2_F_2_–P	0.0158	0.0412	0.0001
CdBr_2_⋯C_2_F_2_–P	0.0168	0.0486	0.0005
HgBr_2_⋯C_2_F_2_–P	0.0140	0.0423	0.0010
ZnBr_2_⋯C_2_Li_2_–P	0.0878	0.2251	−0.0309
CdBr_2_⋯C_2_Li_2_–P	0.0738	0.2095	−0.0189
HgBr_2_⋯C_2_Li_2_–P	0.0869	0.2212	−0.0255

**Table 4:** Electrostatic energy (ES), exchange energy (EX), repulsion energy (REP), polarization energy (POL), and dispersion energy (DISP) in the selected complexes. All are in kcal/mol.

Complexes	ES	EX	REP	POL	DISP
ZnBr_2_⋯C_2_H_2_–P	−31.95	−51.86	96.03	−18.31	−5.66
CdBr_2_⋯C_2_H_2_–P	−23.41	−38.14	69.28	−11.88	−5.65
HgBr_2_⋯C_2_H_2_–P	−10.75	−20.25	35.06	−3.99	−4.70
ZnBr_2_⋯C_2_H_2_–T	−18.09	−34.58	62.46	−11.21	−5.46
CdBr_2_⋯C_2_H_2_–T	−14.02	−27.28	48.73	−8.27	−5.60
HgBr_2_⋯C_2_H_2_–T	−7.14	−17.03	29.18	−3.34	−5.11
ZnBr_2_⋯C_2_F_2_–P	−7.24	−18.32	32.41	−4.09	−5.88
CdBr_2_⋯C_2_F_2_–P	−6.89	−17.67	31.40	−4.21	−6.34
HgBr_2_⋯C_2_F_2_–P	−4.47	−13.30	23.02	−2.17	−6.04
ZnBr_2_⋯C_2_Li_2_–P	−171.59	−175.81	340.82	−86.87	−10.41
CdBr_2_⋯C_2_Li_2_–P	−157.82	−166.35	322.57	−81.82	−11.45
HgBr_2_⋯C_2_Li_2_–P	−169.45	−211.53	410.98	−118.60	−10.72
ZnBr_2_⋯C_2_H_4_–P	−33.38	−57.61	106.78	−20.80	−7.76
CdBr_2_⋯C_2_H_4_–P	−24.55	−43.50	79.30	−14.31	−7.45
HgBr_2_⋯C_2_H_4_–P	−11.32	−24.16	41.93	−4.88	−6.11
ZnBr_2_⋯C_2_H_4_–T	−29.13	−49.86	92.49	−17.69	−7.18
CdBr_2_⋯C_2_H_4_–T	−22.86	−40.02	73.28	−13.14	−7.27
HgBr_2_⋯C_2_H_4_–T	−13.64	−28.38	50.07	−6.44	−6.75

**Table 5:** Binding distance (*R* in Å) and interaction energy (Δ*E* in kcal/mol) for some interactions, including π systems.

Complexes	*R*	Δ*E*	Method	Types	Reference
FH⋯C_2_H_2_	0.931	−2.88	MP2/aug-cc-pVTZ	π–hydrogen	[30]
FH⋯C_2_H_4_	0.932	−2.87	Δ*E*_int(BSSE)_		
FLi⋯C_2_H_2_	2.356	−7.73	MP2/6-311++G(d,p)	π–lithium	[33]
FLi⋯C_2_H_4_	2.325	−7.72	Δ*E*_int(BSSE)_		
FNa⋯C_2_H_2_	2.760	−5.20	MP2/6-311++G(d,p)	π–sodium	[35]
FNa⋯C_2_H_4_	2.808	−5.23	Δ*E*_int(BSSE)_		
F_2_Be⋯C_2_H_2_	-	14.11	CCSD(T)/aug-cc-pVTZ//MP2/aug-cc-pVTZ	π–beryllium	[34]
F_2_Be⋯C_2_H_4_	-	13.16	Δ*E*_CCSD(T)_		
F_2_Mg⋯C_2_H_2_	2.460	−15.00	MP2/aug-cc-pVTZ	π–magnesium	[36]
F_2_Mg⋯C_2_H_4_	2.523	−13.16	Δ*E*_int(BSSE)_		
F_3_Al⋯C_2_H_2_	2.437	−18.7	MP2/aug-cc-pVTZ	π–triel	[41]
F_3_Al⋯C_2_H_4_	2.467	−20.1	Δ*E*_int(BSSE)_		
FH_3_Ge⋯C_2_H_2_	3.299	−2.80	MP2/aug-cc-pVTZ	π–tetrel	[42]
FH_3_Ge⋯C_2_H_4_	3.269	−2.53	Δ*E*_bind(BSSE)_		
FH_2_As⋯C_2_H_2_	3.013	−4.03	MP2/aug-cc-pVTZ	π–pnictogen	[81]
FH_2_As⋯C_2_H_4_	2.907	−4.60	Δ*E*_int(BSSE)_		
F_2_S⋯C_2_H_2_	2.988	3.79	MP2/aug-cc-pVTZ	π–chalcogen	[82]
F_2_S⋯C_2_H_4_	2.904	4.47	Δ*E*_bind(BSSE)_		
FBr⋯C_2_H_2_	2.813	−4.90	CCSD(T)/aug-cc-pVTZ//*ω*B97XD/aug-cc-pVTZ	π–halogen	[83]
FBr⋯C_2_H_4_	2.681	−6.69	Δ*E*_CCSD(T)_		
FAu⋯C_2_H_2_	2.008	−54.30	CCSD(T)/aug-cc-pVTZ//*ω*B97XD/aug-cc-pVTZ	regium–π	[83]
FAu⋯C_2_H_4_	2.017	−58.79	Δ*E*_CCSD(T)_		
F_2_OXe⋯C_2_H_2_	3.073	−6.6	MP2/aug-cc-pVTZ	aerogen–π	[24]
F_2_OXe⋯C_2_H_4_	3.020	−6.2	Δ*E*_int(BSSE)_		
Cl_2_Zn⋯C_2_H_2_–P	2.406	−11.60	MP2/aug-cc-pVTZ	spodium–π	Our results
Cl_2_Zn⋯C_2_H_4_–P	2.391	−11.68	Δ*E*_int(BSSE)_		

Method: calculated level and expression of the strength of the interaction. Δ*E*_int(BSSE)_: The interaction energy corrected for BSSE was calculated as a difference by subtracting the energy sum of the monomers from the total energy of the complex. Δ*E*_bind(BSSE)_: the binding energy corrected for BSSE was derived as the difference in energy between the optimized dimer and the sum of the individual monomers in their optimized geometries. Δ*E*_CCSD(T)_: single-point energies calculations with CCSD(T) were performed to obtain more accurate interaction energies.

**Table 6:** Binding distance (*R* in Å) and interaction energy (Δ*E* in kcal/mol) for some typical examples of spodium bonds.

Lewis Acid	Lewis Base	*R*	Δ*E*	Reference
ZnBr_2_L_2_ (L = thiourea)	CO	3.79	−2.2 ^a^	[1]
CdCl_2_L_2_ (L = thiourea)	H_2_CS	2.95	−8.9 ^a^	
CdCO_3_	NCH	2.146	−31.84 ^b^	[20]
HgCO_3_	NHCH_2_	2.047	−56.68 ^b^	
HgCl_3_^−^	HgCl_3_^−^	3.616	−1.88 ^c^	[18]
ZnMe_2_	cyclopropenylidene	2.192	10.3 ^d^	[15]
ZnF_2_	(NH_3_)_2_C	1.879	78.8 ^d^	
HgI_2_	C_2_F_2_	3.177	−0.79	Our results
ZnCl_2_	C_2_Li_2_	1.965	−106.75	

^a^: RI-MP2/aug-cc-pVTZ-based interaction energies. ^b^: MP2/aug-cc-pVTZ-based BSSE-corrected interaction energies. ^c^: MP2/aug-cc-pVDZ-based BSSE-corrected interaction energies. ^d^: *ω*B97X-D/6-311++G(2df,2p)-based dissociation energies *D*_0_.

## Data Availability

Not applicable.

## Supplementary Materials

The following supporting information can be downloaded at: https://www.mdpi.com/article/10.3390/molecules27092885/s1, Table S1: distances (*R*, Å) between M in MX_2_ and the centers of C–C bond in π molecules (* denote the centers of C–C bond), angles of X-M-X in MX_2_ (α, deg), dihedral angle of X-X⋯C-C (*θ*, deg), and interaction energy (Δ*E*, kcal/mol) in the complexes at the MP2/aug-cc-pVTZ level. Table S2: charge transfer (CT, e), second-order perturbation energies (*E*^(2)^, kcal/mol) and Wiberg bond index (WBI) in the complexes in the complexes at the HF/aug-cc-pVTZ level. Table S3: electron density (*ρ*, au), Laplacian (▽^2^*ρ*, au), and total energy density (*H*, au) at the intermolecular bond critical points (BCPs) in the complexes at the MP2/aug-cc-pVTZ level. Table S4: electrostatic energy (ES), exchange energy (EX), repulsion energy (REP), polarization energy (POL), and dispersion energy (DISP) in the selected complexes at the MP2/aug-cc-pVTZ level. All are in kcal/mol. Figure S1: the optimized geometries of the MX_2_ (M = Zn, Cd, Hg; X = Cl, Br, I), C_2_H_2_, C_2_F_2_, C_2_Li_2_, and C_2_H_4_ molecules at the MP2/aug-cc-pVTZ level. Figure S2: plots of the reduced density gradient RDG versus the electron density multiplied by the sign of the second Hessian eigenvalue (sign(λ_2_)*ρ*) of MX_2_⋯C_2_H_2_–P. NCI maps of the corresponding binary complexes. Blue, green, orange, and red areas correspond to strong attractive, weak attractive, weak repulsive, and strong repulsive interactions, respectively. Figure S3: plots of the reduced density gradient RDG versus the electron density multiplied by the sign of the second Hessian eigenvalue (sign(λ_2_)*ρ*) of MX_2_⋯C_2_H_2_–T. NCI maps of the corresponding binary complexes. Figure S4: plots of the reduced density gradient RDG versus the electron density multiplied by the sign of the second Hessian eigenvalue (sign(λ_2_)*ρ*) of MX_2_⋯C_2_H_4_–P. NCI maps of the corresponding binary complexes. Figure S5: plots of the reduced density gradient RDG versus the electron density multiplied by the sign of the second Hessian eigenvalue (sign(λ_2_)*ρ*) of MX_2_⋯C_2_H_4_–T. NCI maps of the corresponding binary complexes. Figure S6: plots of the reduced density gradient RDG versus the electron density multiplied by the sign of the second Hessian eigenvalue (sign(λ_2_)*ρ*) of MX_2_⋯C_2_F_2_–T. NCI maps of the corresponding binary complexes. Figure S7: Pplots of the reduced density gradient RDG versus the electron density multiplied by the sign of the second Hessian eigenvalue (sign(λ_2_)*ρ*) of MX_2_⋯C_2_Li_2_–T. NCI maps of the corresponding binary complexes.Figure S8: molecular graphs of spodium–π bonded stable complexes at the intermolecular bond critical points. Figure S9: electron density shifts of the MX_2_⋯C_2_H_2_–P complexes (iso = ±0.0008). Red regions indicate increased electron density, while blue regions represent decreased electron density. Figure S10: electron density shifts of the MX_2_⋯C_2_H_2_–T complexes. Figure S11: electron density shifts of the MX_2_⋯C_2_H_4_–P complexes. Figure S12: Electron density shifts of the MX_2_⋯C_2_H_4_-T complexes. Figure S13: electron density shifts of the MX_2_⋯C_2_F_2_–P complexes. Figure S14: electron density shifts of the MX_2_⋯C_2_Li_2_–P complexes. Figure S15: comparison of electrostatic, polarization, and dispersion energies in the binary complexes.

## Sample Availability

Samples of the compounds are not available from the authors.
