# Supplementary material for: Insight into Spodium–π Bonding Characteristics of the MX2⋯π (M = Zn, Cd and Hg; X = Cl, Br and I) Complexes—A Theoretical Study
Source: Molecules. 2022 Apr 30;27(9):2885. doi: 10.3390/molecules27092885 (PMC9101229; doi:10.3390/molecules27092885)
Supplement: Supplementary file 1 [file molecules-27-02885-s001.zip › molecules-1670722-supplementary.pdf]

# Supporting Information

## Insight into spodium–π bonding characteristics of the $MX_2\cdots\pi$ (M = Zn, Cd and Hg; X = Cl, Br and I) complexes–A theoretical study

Meng Gao [1,*], Qibo Zhao [1], Hao Yu [1], Min Fu [1], and Qingzhong Li [2,*]

[1] College of Chemical and Biological Engineering, Shandong University of Science and Technology, Qingdao 266590, China;

[2] The Laboratory of Theoretical and Computational Chemistry, School of Chemistry and Chemical Engineering, Yantai University, Yantai 264005, China

***** Corresponding Authors: Meng Gao, E-mail: skd996370@sdust.edu.cn; Qingzhong Li, E-mail: li70316@sohu.com.

**Table S1.** Distances ($R$, Å) between M in $MX_2$ and the centers of C–C bond in $\pi$ molecules ($^*$ denote the centers of C–C bond), angles of X-M-X in $MX_2$ ($\alpha$, deg), dihedral angle of X–X···C–C ($\theta$, deg), and interaction energy ($\Delta E$, kcal/mol) in the complexes at the MP2/aug-cc-pVTZ level.

| | $R_{M\cdots *}$ | $R_{M\text{-}X}$ | $R_{C\text{-}C}$ | $\alpha$ | $\theta$ | $\Delta E$ |
|---|---|---|---|---|---|---|
| $ZnCl_2\cdots C_2H_2$–P | 2.406 | 2.094 | 1.218 | 151 | 0 | –11.60 |
| $ZnBr_2\cdots C_2H_2$–P | 2.387 | 2.222 | 1.219 | 149 | 0 | –9.72 |
| $ZnI_2\cdots C_2H_2$–P | 2.387 | 2.411 | 1.220 | 146 | 0 | –8.30 |
| $CdCl_2\cdots C_2H_2$–P | 2.664 | 2.280 | 1.218 | 163 | 0 | –9.66 |
| $CdBr_2\cdots C_2H_2$–P | 2.670 | 2.401 | 1.218 | 161 | 0 | –8.26 |
| $CdI_2\cdots C_2H_2$–P | 2.700 | 2.580 | 1.218 | 159 | 0 | –6.82 |
| $HgCl_2\cdots C_2H_2$–P | 3.056 | 2.238 | 1.215 | 176 | 0 | –3.92 |
| $HgBr_2\cdots C_2H_2$–P | 3.078 | 2.363 | 1.215 | 175 | 0 | –2.96 |
| $HgI_2\cdots C_2H_2$–P | 3.145 | 2.541 | 1.215 | 175 | 0 | –2.11 |
| $ZnCl_2\cdots C_2H_2$–T | 2.545 | 2.077 | 1.216 | 159 | 83 | –6.26 |
| $ZnBr_2\cdots C_2H_2$–T | 2.538 | 2.204 | 1.216 | 158 | 83 | –5.29 |
| $ZnI_2\cdots C_2H_2$–T | 2.575 | 2.391 | 1.216 | 157 | 83 | –4.76 |
| $CdCl_2\cdots C_2H_2$–T | 2.757 | 2.268 | 1.216 | 166 | 83 | –6.11 |
| $CdBr_2\cdots C_2H_2$–T | 2.770 | 2.389 | 1.216 | 165 | 83 | –5.24 |
| $CdI_2\cdots C_2H_2$–T | 2.806 | 2.566 | 1.216 | 165 | 83 | –4.31 |
| $HgCl_2\cdots C_2H_2$–T | 3.083 | 2.234 | 1.214 | 177 | 84 | –2.30 |
| $HgBr_2\cdots C_2H_2$–T | 3.081 | 2.359 | 1.214 | 176 | 84 | –1.78 |
| $HgI_2\cdots C_2H_2$–T | 3.127 | 2.541 | 1.214 | 176 | 83 | –1.37 |
| $ZnCl_2\cdots C_2H_4$–P | 2.391 | 2.090 | 1.346 | 148 | 0 | –11.68 |
| $ZnBr_2\cdots C_2H_4$–P | 2.367 | 2.225 | 1.347 | 146 | 0 | –10.89 |
| $ZnI_2\cdots C_2H_4$–P | 2.361 | 2.414 | 1.348 | 143 | 0 | –9.78 |
| $CdCl_2\cdots C_2H_4$–P | / | / | / | / | / | / |
| $CdBr_2\cdots C_2H_4$–P | 2.635 | 2.406 | 1.344 | 157 | 0 | –8.58 |
| $CdI_2\cdots C_2H_4$–P | 2.649 | 2.584 | 1.345 | 155 | 0 | –7.26 |
| $HgCl_2\cdots C_2H_4$–P | / | / | / | / | / | / |
| $HgBr_2\cdots C_2H_4$–P | 3.051 | 2.364 | 1.338 | 174 | 0 | –2.12 |
| $HgI_2\cdots C_2H_4$–P | 3.090 | 2.542 | 1.338 | 173 | 0 | –1.40 |
| $ZnCl_2\cdots C_2H_4$–T | 2.410 | 2.089 | 1.346 | 155 | 82 | –11.09 |
| $ZnBr_2\cdots C_2H_4$–T | 2.400 | 2.216 | 1.346 | 153 | 82 | –9.51 |

| | | | | | | |
|---|---|---|---|---|---|---|
| $ZnI_2\cdots C_2H_4$–T | 2.409 | 2.405 | 1.346 | 152 | 82 | –7.87 |
| $CdCl_2\cdots C_2H_4$–T | 2.637 | 2.279 | 1.344 | 163 | 82 | –9.52 |
| $CdBr_2\cdots C_2H_4$–T | 2.640 | 2.401 | 1.344 | 163 | 82 | –8.17 |
| $CdI_2\cdots C_2H_4$–T | 2.664 | 2.578 | 1.344 | 162 | 82 | –6.80 |
| $HgCl_2\cdots C_2H_4$–T | 2.934 | 2.241 | 1.339 | 175 | 82 | –3.32 |
| $HgBr_2\cdots C_2H_4$–T | 2.924 | 2.365 | 1.339 | 175 | 82 | –2.30 |
| $HgI_2\cdots C_2H_4$–T | 2.949 | 2.543 | 1.339 | 174 | 82 | –1.60 |
| $ZnCl_2\cdots C_2F_2$–P | 2.869 | 2.058 | 1.199 | 171 | 33 | –2.36 |
| $ZnBr_2\cdots C_2F_2$–P | 2.886 | 2.184 | 1.199 | 171 | 42 | –1.26 |
| $ZnI_2\cdots C_2F_2$–P | 2.958 | 2.372 | 1.198 | 172 | 50 | –1.36 |
| $CdCl_2\cdots C_2F_2$–P | 2.957 | 2.255 | 1.200 | 173 | 33 | –3.25 |
| $CdBr_2\cdots C_2F_2$–P | 2.976 | 2.376 | 1.199 | 174 | 40 | –2.39 |
| $CdI_2\cdots C_2F_2$–P | 3.026 | 2.554 | 1.199 | 174 | 47 | –1.77 |
| $HgCl_2\cdots C_2F_2$–P | 3.162 | 2.230 | 1.197 | 179 | 39 | –1.64 |
| $HgBr_2\cdots C_2F_2$–P | 3.152 | 2.355 | 1.197 | 179 | 46 | –1.03 |
| $HgI_2\cdots C_2F_2$–P | 3.177 | 2.533 | 1.197 | 179 | 52 | –0.79 |
| $ZnCl_2\cdots C_2Li_2$–P | 1.965 | 2.222 | 1.277 | 120 | 0 | –106.75 |
| $ZnBr_2\cdots C_2Li_2$–P | 1.968 | 2.348 | 1.276 | 119 | 0 | –99.94 |
| $ZnI_2\cdots C_2Li_2$–P | 1.976 | 2.535 | 1.276 | 117 | 1 | –93.57 |
| $CdCl_2\cdots C_2Li_2$–P | 2.166 | 2.421 | 1.279 | 134 | 0 | –97.43 |
| $CdBr_2\cdots C_2Li_2$–P | 2.172 | 2.534 | 1.279 | 132 | 0 | –89.97 |
| $CdI_2\cdots C_2Li_2$–P | 2.180 | 2.709 | 1.278 | 128 | 0 | –83.63 |
| $HgCl_2\cdots C_2Li_2$–P | 2.141 | 2.439 | 1.291 | 129 | 0 | –96.58 |
| $HgBr_2\cdots C_2Li_2$–P | 2.152 | 2.543 | 1.289 | 126 | 0 | –87.51 |
| $HgI_2\cdots C_2Li_2$–P | 2.167 | 2.699 | 1.287 | 123 | 0 | –78.82 |

**Table S2.** Charge transfer (CT, e), Second-order perturbation energies ($E^{(2)}$, kcal/mol) and Wiberg bond index (WBI) in the complexes at the HF/aug-cc-pVTZ level.

| | CT | $E^{(2)}$ | WBI |
|---|---|---|---|
| $ZnCl_2\cdots C_2H_2$–P | –0.067 | 35.25 | 0.1018 |
| $ZnBr_2\cdots C_2H_2$–P | –0.073 | 46.63 | 0.1108 |
| $ZnI_2\cdots C_2H_2$–P | –0.074 | 39.06 | 0.1149 |
| $CdCl_2\cdots C_2H_2$–P | –0.061 | 24.99 | 0.0690 |
| $CdBr_2\cdots C_2H_2$–P | –0.067 | 27.38 | 0.0738 |
| $CdI_2\cdots C_2H_2$–P | –0.068 | 27.80 | 0.0752 |
| $HgCl_2\cdots C_2H_2$–P | –0.022 | 7.58 | 0.0280 |
| $HgBr_2\cdots C_2H_2$–P | –0.024 | 8.63 | 0.0309 |
| $HgI_2\cdots C_2H_2$–P | –0.023 | 8.19 | 0.0317 |
| $ZnCl_2\cdots C_2H_2$–T | –0.063 | 30.70 | 0.0823 |
| $ZnBr_2\cdots C_2H_2$–T | –0.071 | 34.32 | 0.0760 |
| $ZnI_2\cdots C_2H_2$–T | –0.074 | 31.94 | 0.0701 |
| $CdCl_2\cdots C_2H_2$–T | –0.060 | 20.95 | 0.0571 |
| $CdBr_2\cdots C_2H_2$–T | –0.067 | 22.56 | 0.0612 |
| $CdI_2\cdots C_2H_2$–T | –0.069 | 22.14 | 0.0615 |
| $HgCl_2\cdots C_2H_2$–T | –0.025 | 7.62 | 0.0271 |
| $HgBr_2\cdots C_2H_2$–T | –0.030 | 8.78 | 0.0309 |
| $HgI_2\cdots C_2H_2$–T | –0.033 | 9.50 | 0.0331 |
| $ZnCl_2\cdots C_2H_4$–P | –0.078 | 27.50 | 0.0920 |
| $ZnBr_2\cdots C_2H_4$–P | –0.082 | 44.90 | 0.0996 |
| $ZnI_2\cdots C_2H_4$–P | –0.083 | 57.23 | 0.1044 |
| $CdCl_2\cdots C_2H_4$–P | / | / | / |
| $CdBr_2\cdots C_2H_4$–P | –0.098 | 31.43 | 0.0981 |
| $CdI_2\cdots C_2H_4$–P | –0.099 | 33.48 | 0.0999 |
| $HgCl_2\cdots C_2H_4$–P | / | / | / |
| $HgBr_2\cdots C_2H_4$–P | –0.039 | 12.95 | 0.0438 |
| $HgI_2\cdots C_2H_4$–P | –0.040 | 13.40 | 0.0450 |
| $ZnCl_2\cdots C_2H_4$–T | –0.085 | 44.95 | 0.0851 |
| $ZnBr_2\cdots C_2H_4$–T | –0.093 | 48.67 | 0.0907 |
| $ZnI_2\cdots C_2H_4$–T | –0.100 | 58.39 | 0.0934 |
| $CdCl_2\cdots C_2H_4$–T | –0.095 | 27.99 | 0.0883 |
| $CdBr_2\cdots C_2H_4$–T | –0.102 | 30.13 | 0.0922 |

| | | | |
|---|---|---|---|
| $CdI_2\cdots C_2H_4$–T | –0.106 | 31.59 | 0.0923 |
| $HgCl_2\cdots C_2H_4$–T | –0.048 | 14.69 | 0.0484 |
| $HgBr_2\cdots C_2H_4$–T | –0.055 | 17.06 | 0.0539 |
| $HgI_2\cdots C_2H_4$–T | –0.059 | 17.98 | 0.0556 |
| $ZnCl_2\cdots C_2F_2$–P | –0.039 | 15.43 | 0.0412 |
| $ZnBr_2\cdots C_2F_2$–P | –0.042 | 17.84 | 0.0433 |
| $ZnI_2\cdots C_2F_2$–P | –0.041 | 18.9 | 0.0407 |
| $CdCl_2\cdots C_2F_2$–P | –0.040 | 13.89 | 0.0414 |
| $CdBr_2\cdots C_2F_2$–P | –0.043 | 15.31 | 0.0430 |
| $CdI_2\cdots C_2F_2$–P | –0.042 | 15.88 | 0.0436 |
| $HgCl_2\cdots C_2F_2$–P | –0.020 | 7.07 | 0.0231 |
| $HgBr_2\cdots C_2F_2$–P | –0.024 | 8.93 | 0.0267 |
| $HgI_2\cdots C_2F_2$–P | –0.026 | 10.53 | 0.0290 |
| $ZnCl_2\cdots C_2Li_2$–P | –0.907 | 128.48 | 0.2540 |
| $ZnBr_2\cdots C_2Li_2$–P | –1.043 | 61.84 | 0.2619 |
| $ZnI_2\cdots C_2Li_2$–P | –0.870 | 24.99 | 0.2758 |
| $CdCl_2\cdots C_2Li_2$–P | –1.090 | 113.82 | 0.2967 |
| $CdBr_2\cdots C_2Li_2$–P | –1.094 | 110.02 | 0.3052 |
| $CdI_2\cdots C_2Li_2$–P | –1.055 | 107.76 | 0.3098 |
| $HgCl_2\cdots C_2Li_2$–P | –1.293 | 48.5(91.98)[a] | 0.3873 |
| $HgBr_2\cdots C_2Li_2$–P | –1.157 | 183.55 | 0.3881 |
| $HgI_2\cdots C_2Li_2$–P | –1.040 | 171.21 | 0.3876 |

Note: The charge transfer is the sum of atomic charge of the $MX_2$ in the complexes. $E^{(2)}$ is the stabilization energy due to the orbital interactions of $BD_{C=C/C\equiv C}\rightarrow LP^*_M$. [a] the value in the parentheses is the stabilization energy due to the orbital interactions of $BD_{C-Li}\rightarrow LP^*_M$. WBI values correspond to the M···C in the complexes.

**Table S3.** Electron density (ρ, au), Laplacian ($\nabla^2\rho$, au), and total energy density ($H$, au) at the intermolecular bond critical points (BCPs) in the complexes at the MP2/aug-cc-pVTZ level.

| | ρ | $\nabla^2\rho$ | *H* |
|---|---|---|---|
| $ZnCl_2\cdots C_2H_2$–P | 0.0384 | 0.0988 | –0.0070 |
| $ZnBr_2\cdots C_2H_2$–P | 0.0398 | 0.1023 | –0.0076 |
| $ZnI_2\cdots C_2H_2$–P | 0.0400 | 0.1018 | –0.0077 |
| $CdCl_2\cdots C_2H_2$–P | 0.0295 | 0.0875 | –0.0018 |
| $CdBr_2\cdots C_2H_2$–P | 0.0292 | 0.0863 | –0.0018 |
| $CdI_2\cdots C_2H_2$–P | 0.0276 | 0.0812 | –0.0014 |
| $HgCl_2\cdots C_2H_2$–P | 0.0166 | 0.0499 | 0.0008 |
| $HgBr_2\cdots C_2H_2$–P | 0.0160 | 0.0480 | 0.0009 |
| $HgI_2\cdots C_2H_2$–P | 0.0141 | 0.0427 | 0.0010 |
| $ZnCl_2\cdots C_2H_2$–T | 0.0292 | 0.0747 | –0.0036 |
| $ZnBr_2\cdots C_2H_2$–T | 0.0297 | 0.0754 | –0.0037 |
| $ZnI_2\cdots C_2H_2$–T | 0.0283 | 0.0711 | –0.0033 |
| $CdCl_2\cdots C_2H_2$–T | 0.0245 | 0.0722 | –0.0006 |
| $CdBr_2\cdots C_2H_2$–T | 0.0242 | 0.0708 | –0.0006 |
| $CdI_2\cdots C_2H_2$–T | 0.0226 | 0.0655 | –0.0003 |
| $HgCl_2\cdots C_2H_2$–T | 0.0159 | 0.0473 | 0.0009 |
| $HgBr_2\cdots C_2H_2$–T | 0.0157 | 0.0468 | 0.0009 |
| $HgI_2\cdots C_2H_2$–T | 0.0147 | 0.0438 | 0.0010 |
| $ZnCl_2\cdots C_2H_4$–P | 0.0402 | 0.0857 | –0.0083 |
| $ZnBr_2\cdots C_2H_4$–P | 0.0421 | 0.0893 | –0.0091 |
| $ZnI_2\cdots C_2H_4$–P | 0.0426 | 0.0898 | –0.0094 |
| $CdCl_2\cdots C_2H_4$–P | / | / | / |
| $CdBr_2\cdots C_2H_4$–P | 0.0319 | 0.0817 | –0.0030 |
| $CdI_2\cdots C_2H_4$–P | 0.0311 | 0.0793 | –0.0028 |
| $HgCl_2\cdots C_2H_4$–P | / | / | / |
| $HgBr_2\cdots C_2H_4$–P | 0.0175 | 0.0463 | 0.0005 |
| $HgI_2\cdots C_2H_4$–P | 0.0163 | 0.0434 | 0.0006 |
| $ZnCl_2\cdots C_2H_4$–T | 0.0385 | 0.0832 | –0.0075 |
| $ZnBr_2\cdots C_2H_4$–T | 0.0393 | 0.0843 | –0.0078 |
| $ZnI_2\cdots C_2H_4$–T | 0.0388 | 0.0821 | –0.0077 |

| | | | |
|---|---|---|---|
| $CdCl_2\cdots C_2H_4$–T | 0.0316 | 0.0814 | –0.0028 |
| $CdBr_2\cdots C_2H_4$–T | 0.0315 | 0.0807 | –0.0028 |
| $CdI_2\cdots C_2H_4$–T | 0.0302 | 0.0770 | –0.0025 |
| $HgCl_2\cdots C_2H_4$–T | 0.0215 | 0.0561 | –0.0002 |
| $HgBr_2\cdots C_2H_4$–T | 0.0220 | 0.0570 | –0.0003 |
| $HgI_2\cdots C_2H_4$–T | 0.0211 | 0.0547 | –0.0001 |
| $ZnCl_2\cdots C_2F_2$–P | 0.0162 | 0.0426 | 0.0000 |
| $ZnBr_2\cdots C_2F_2$–P | 0.0158 | 0.0412 | 0.0001 |
| $ZnI_2\cdots C_2F_2$–P | 0.0140 | 0.0363 | 0.0003 |
| $CdCl_2\cdots C_2F_2$–P | 0.0173 | 0.0506 | 0.0005 |
| $CdBr_2\cdots C_2F_2$–P | 0.0168 | 0.0486 | 0.0005 |
| $CdI_2\cdots C_2F_2$–P | 0.0154 | 0.0440 | 0.0006 |
| $HgCl_2\cdots C_2F_2$–P | 0.0143 | 0.0430 | 0.0010 |
| $HgBr_2\cdots C_2F_2$–P | 0.0140 | 0.0423 | 0.0010 |
| $HgI_2\cdots C_2F_2$–P | 0.0138 | 0.0413 | 0.0010 |
| $ZnCl_2\cdots C_2Li_2$–P | 0.0882 | 0.2278 | –0.0312 |
| $ZnBr_2\cdots C_2Li_2$–P | 0.0878 | 0.2251 | –0.0309 |
| $ZnI_2\cdots C_2Li_2$–P | 0.0867 | 0.2204 | –0.0303 |
| $CdCl_2\cdots C_2Li_2$–P | 0.0746 | 0.2134 | –0.0192 |
| $CdBr_2\cdots C_2Li_2$–P | 0.0738 | 0.2095 | –0.0189 |
| $CdI_2\cdots C_2Li_2$–P | 0.0729 | 0.2050 | –0.0185 |
| $HgCl_2\cdots C_2Li_2$–P | 0.0883 | 0.2272 | –0.0261 |
| $HgBr_2\cdots C_2Li_2$–P | 0.0869 | 0.2212 | –0.0255 |
| $HgI_2\cdots C_2Li_2$–P | 0.0849 | 0.2132 | –0.0245 |

**Table S4.** Electrostatic energy (ES), exchange energy (EX), repulsion energy (REP), polarization energy (POL), and dispersion energy (DISP) in the selected complexes at the MP2/aug-cc-pVTZ level. All are in kcal/mol.

| | ES | EX | REP | POL | DISP |
|---|---|---|---|---|---|
| $ZnCl_2\cdots C_2H_2$–P | –29.51 | –45.06 | 83.67 | –16.43 | –4.99 |
| $ZnBr_2\cdots C_2H_2$–P | –31.95 | –51.86 | 96.03 | –18.31 | –5.66 |
| $ZnI_2\cdots C_2H_2$–P | –33.54 | –58.16 | 106.98 | –20.00 | –6.13 |
| $CdCl_2\cdots C_2H_2$–P | –22.82 | –34.95 | 63.84 | –11.36 | –5.33 |
| $CdBr_2\cdots C_2H_2$–P | –23.41 | –38.14 | 69.28 | –11.88 | –5.65 |
| $CdI_2\cdots C_2H_2$–P | –23.12 | –40.43 | 72.75 | –12.03 | –5.80 |
| $HgCl_2\cdots C_2H_2$–P | –10.79 | –18.85 | 32.79 | –3.95 | –4.35 |
| $HgBr_2\cdots C_2H_2$–P | –10.75 | –20.25 | 35.06 | –3.99 | –4.70 |
| $HgI_2\cdots C_2H_2$–P | –9.88 | –20.48 | 35.07 | –3.72 | –4.97 |
| $ZnCl_2\cdots C_2H_2$–T | –16.81 | –30.52 | 55.49 | –10.42 | –5.00 |
| $ZnBr_2\cdots C_2H_2$–T | –18.09 | –34.58 | 62.46 | –11.21 | –5.46 |
| $ZnI_2\cdots C_2H_2$–T | –18.26 | –37.43 | 66.72 | –11.32 | –5.73 |
| $CdCl_2\cdots C_2H_2$–T | –13.59 | –25.14 | 45.28 | –8.09 | –5.36 |
| $CdBr_2\cdots C_2H_2$–T | –14.02 | –27.28 | 48.73 | –8.27 | –5.60 |
| $CdI_2\cdots C_2H_2$–T | –13.76 | –28.69 | 50.53 | –8.00 | –5.70 |
| $HgCl_2\cdots C_2H_2$–T | –6.91 | –15.60 | 26.93 | –3.27 | –4.78 |
| $HgBr_2\cdots C_2H_2$–T | –7.14 | –17.03 | 29.18 | –3.34 | –5.11 |
| $HgI_2\cdots C_2H_2$–T | –7.14 | –17.03 | 29.18 | –3.34 | –5.11 |
| $ZnCl_2\cdots C_2H_4$–P | –30.23 | –49.51 | 92.10 | –18.56 | –6.91 |
| $ZnBr_2\cdots C_2H_4$–P | –33.38 | –57.61 | 106.78 | –20.80 | –7.76 |
| $ZnI_2\cdots C_2H_4$–P | –35.71 | –65.34 | 120.17 | –22.91 | –8.38 |
| $CdCl_2\cdots C_2H_4$–P | / | / | / | / | / |
| $CdBr_2\cdots C_2H_4$–P | –24.55 | –43.50 | 79.30 | –14.31 | –7.45 |
| $CdI_2\cdots C_2H_4$–P | –25.25 | –47.68 | 86.04 | –14.91 | –7.73 |
| $HgCl_2\cdots C_2H_4$–P | / | / | / | / | / |
| $HgBr_2\cdots C_2H_4$–P | –11.32 | –24.16 | 41.93 | –4.88 | –6.11 |
| $HgI_2\cdots C_2H_4$–P | –11.06 | –25.67 | 44.00 | –4.81 | –6.52 |
| $ZnCl_2\cdots C_2H_4$–T | –27.21 | –44.05 | 82.25 | –16.39 | –6.56 |
| $ZnBr_2\cdots C_2H_4$–T | –29.13 | –49.86 | 92.49 | –17.69 | –7.18 |

| | | | | | |
|---|---|---|---|---|---|
| $ZnI_2\cdots C_2H_4$–T | –30.19 | –55.15 | 101.17 | –18.76 | –7.57 |
| $CdCl_2\cdots C_2H_4$–T | –22.12 | –36.78 | 67.86 | –12.68 | –6.95 |
| $CdBr_2\cdots C_2H_4$–T | –22.86 | –40.02 | 73.28 | –13.14 | –7.27 |
| $CdI_2\cdots C_2H_4$–T | –23.00 | –43.02 | 77.81 | –13.28 | –7.45 |
| $HgCl_2\cdots C_2H_4$–T | –12.84 | –25.36 | 44.94 | –6.00 | –6.24 |
| $HgBr_2\cdots C_2H_4$–T | –13.64 | –28.38 | 50.07 | –6.44 | –6.75 |
| $HgI_2\cdots C_2H_4$–T | –13.47 | –30.12 | 52.52 | –6.40 | –7.14 |
| $ZnCl_2\cdots C_2F_2$–P | –6.91 | –16.31 | 29.05 | –4.12 | –5.24 |
| $ZnBr_2\cdots C_2F_2$–P | –7.24 | –18.32 | 32.41 | –4.09 | –5.88 |
| $ZnI_2\cdots C_2F_2$–P | –6.94 | –19.01 | 33.17 | –3.60 | –6.30 |
| $CdCl_2\cdots C_2F_2$–P | –6.60 | –16.13 | 28.85 | –4.32 | –5.88 |
| $CdBr_2\cdots C_2F_2$–P | –6.89 | –17.67 | 31.40 | –4.21 | –6.34 |
| $CdI_2\cdots C_2F_2$–P | –6.94 | –18.76 | 32.97 | –3.85 | –6.71 |
| $HgCl_2\cdots C_2F_2$–P | –3.99 | –11.54 | 20.02 | –2.08 | –5.44 |
| $HgBr_2\cdots C_2F_2$–P | –4.47 | –13.30 | 23.02 | –2.17 | –6.04 |
| $HgI_2\cdots C_2F_2$–P | –4.82 | –14.77 | 25.39 | –2.11 | –6.58 |
| $ZnCl_2\cdots C_2Li_2$–P | –176.29 | –169.28 | 331.47 | –83.85 | –10.44 |
| $ZnBr_2\cdots C_2Li_2$–P | –171.59 | –175.81 | 340.82 | –86.87 | –10.41 |
| $ZnI_2\cdots C_2Li_2$–P | –161.80 | –182.77 | 347.82 | –91.36 | –9.94 |
| $CdCl_2\cdots C_2Li_2$–P | –164.34 | –163.47 | 319.89 | –80.70 | –11.66 |
| $CdBr_2\cdots C_2Li_2$–P | –157.82 | –166.35 | 322.57 | –81.82 | –11.45 |
| $CdI_2\cdots C_2Li_2$–P | –148.17 | –169.49 | 323.53 | –83.97 | –10.81 |
| $HgCl_2\cdots C_2Li_2$–P | –177.93 | –210.50 | 411.49 | –119.31 | –11.32 |
| $HgBr_2\cdots C_2Li_2$–P | –169.45 | –211.53 | 410.98 | –118.60 | –10.72 |
| $HgI_2\cdots C_2Li_2$–P | –156.48 | –211.24 | 405.84 | –117.79 | –9.73 |

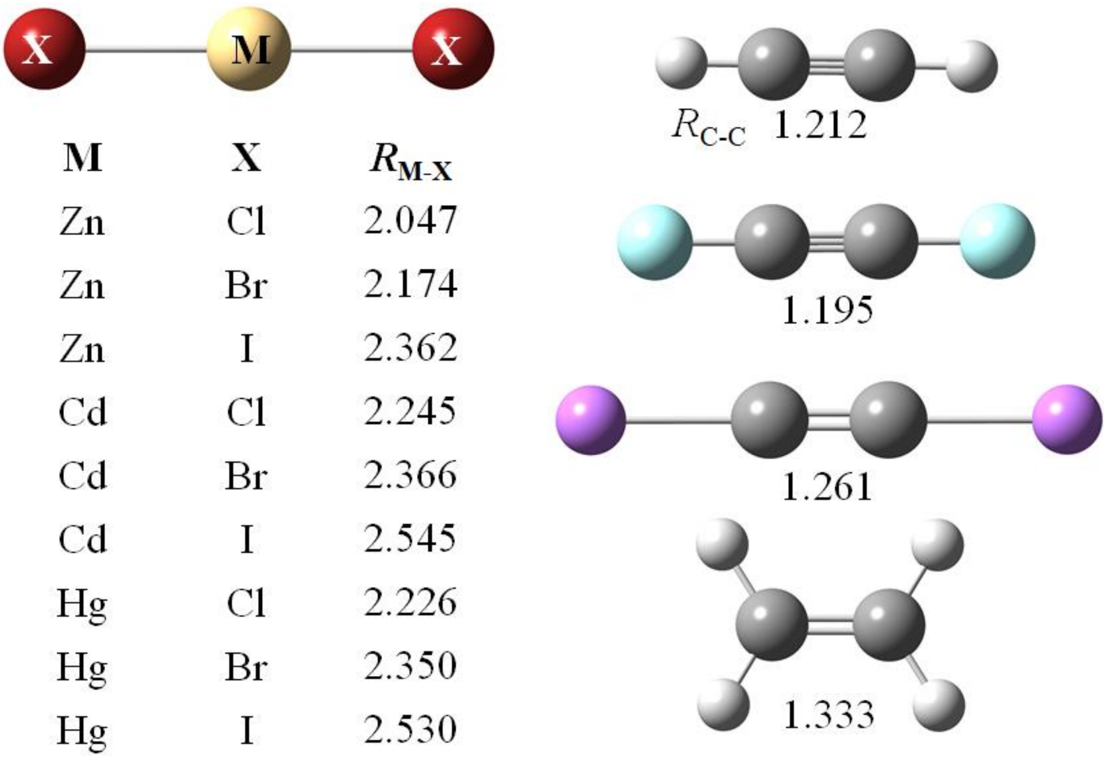


**Figure S1.** The optimized geometries of the $MX_2$ (M=Zn, Cd, Hg; X = Cl, Br, I), $C_2H_2$, $C_2F_2$, $C_2Li_2$ and $C_2H_4$ molecules at the MP2/aug-cc-pVTZ level.

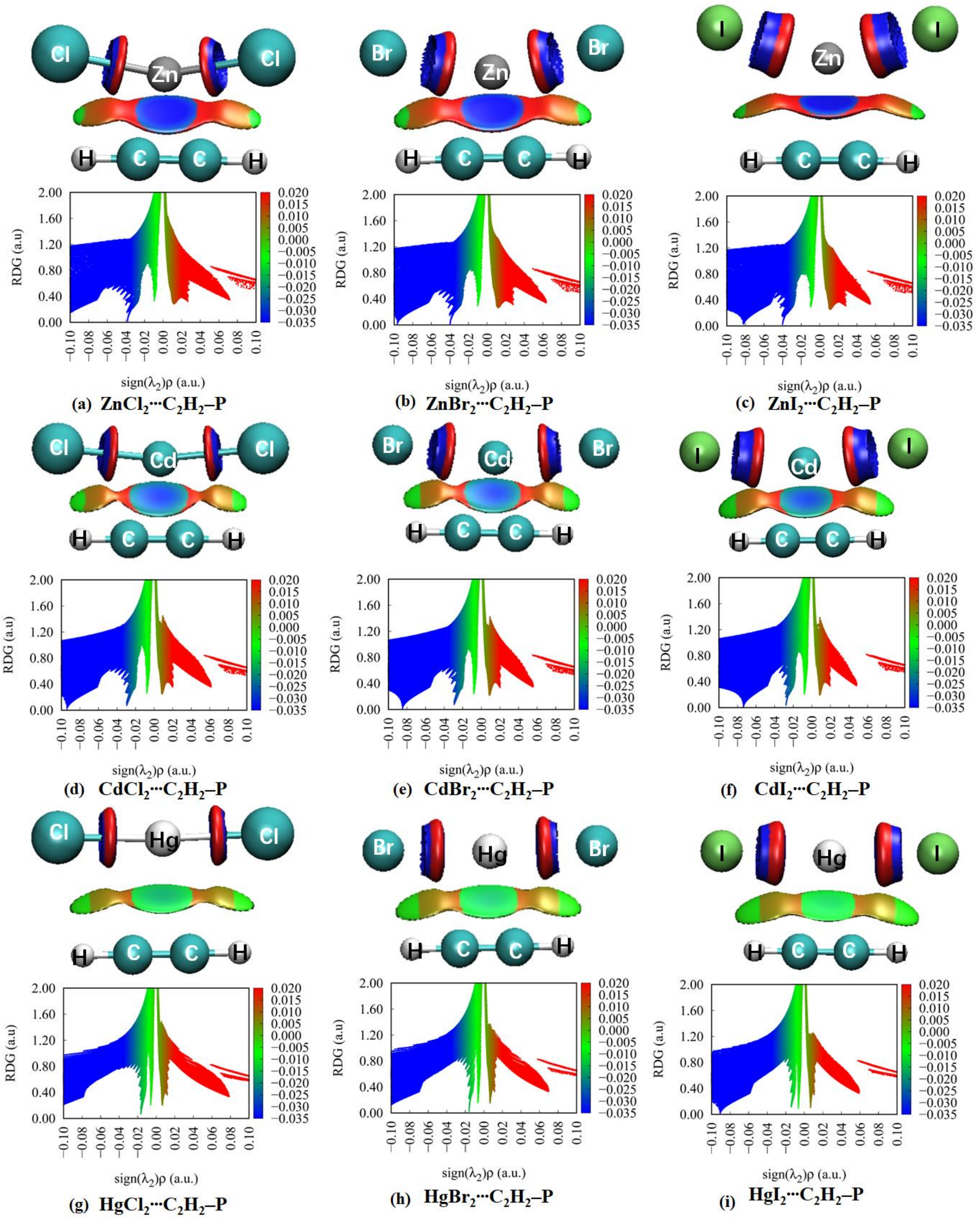


**Figure S2.** Plots of the reduced density gradient RDG *versus* the electron density multiplied by the sign of the second Hessian eigenvalue (sign($\lambda_2$)ρ) of $MX_2\cdots C_2H_2$–P. NCI maps of the corresponding binary complexes. Blue, green, orange, and red areas correspond to strong attractive, weak attractive, weak repulsive, and strong repulsive interactions, respectively.

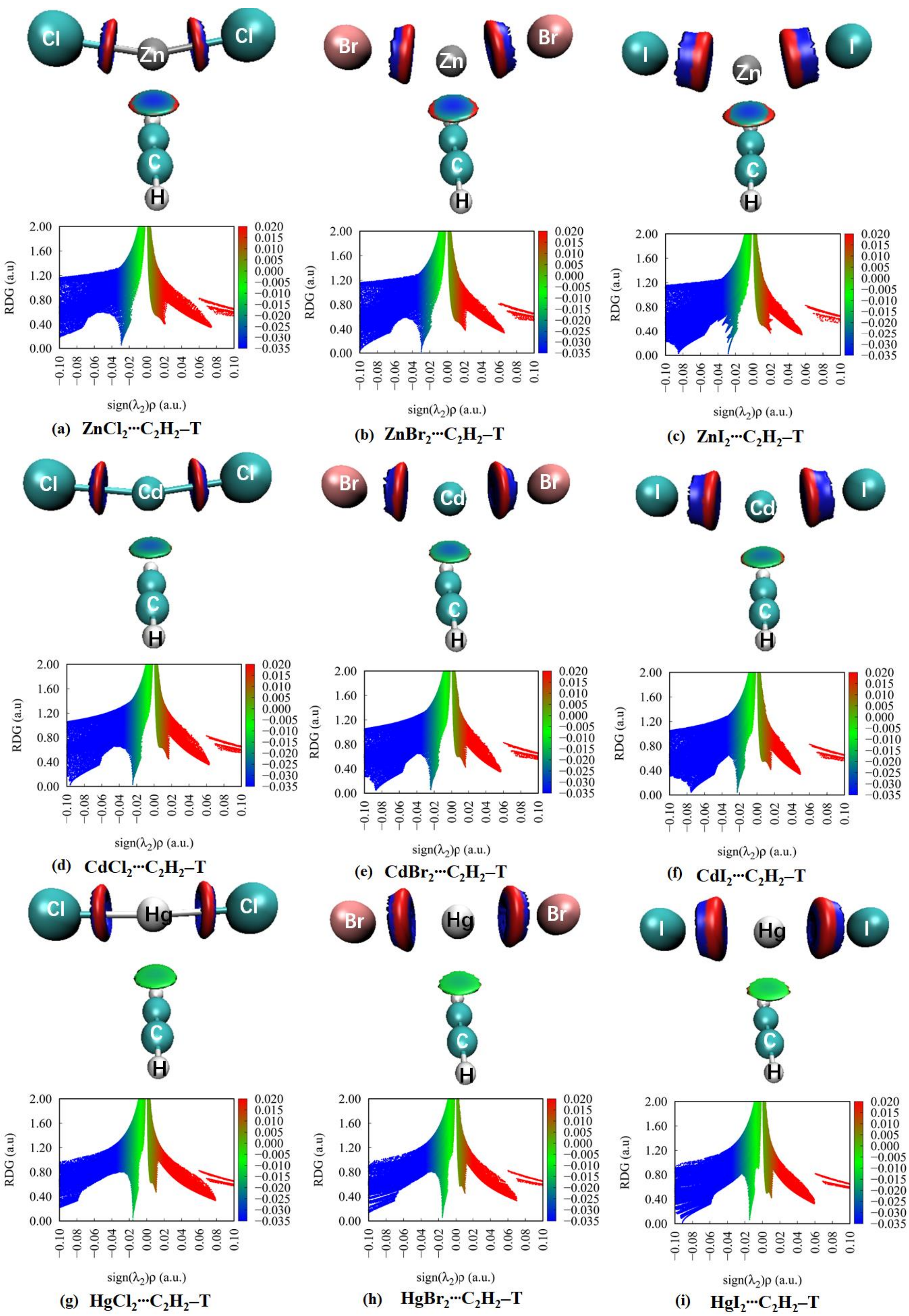


**Figure S3.** Plots of the reduced density gradient RDG *versus* the electron density multiplied by the sign of the second Hessian eigenvalue (sign($\lambda_2$)$\rho$) of $MX_2\cdots C_2H_2$–T. NCI maps of the corresponding binary complexes. Blue, green, orange, and red areas correspond to strong attractive, weak attractive, weak repulsive, and strong repulsive interactions, respectively.

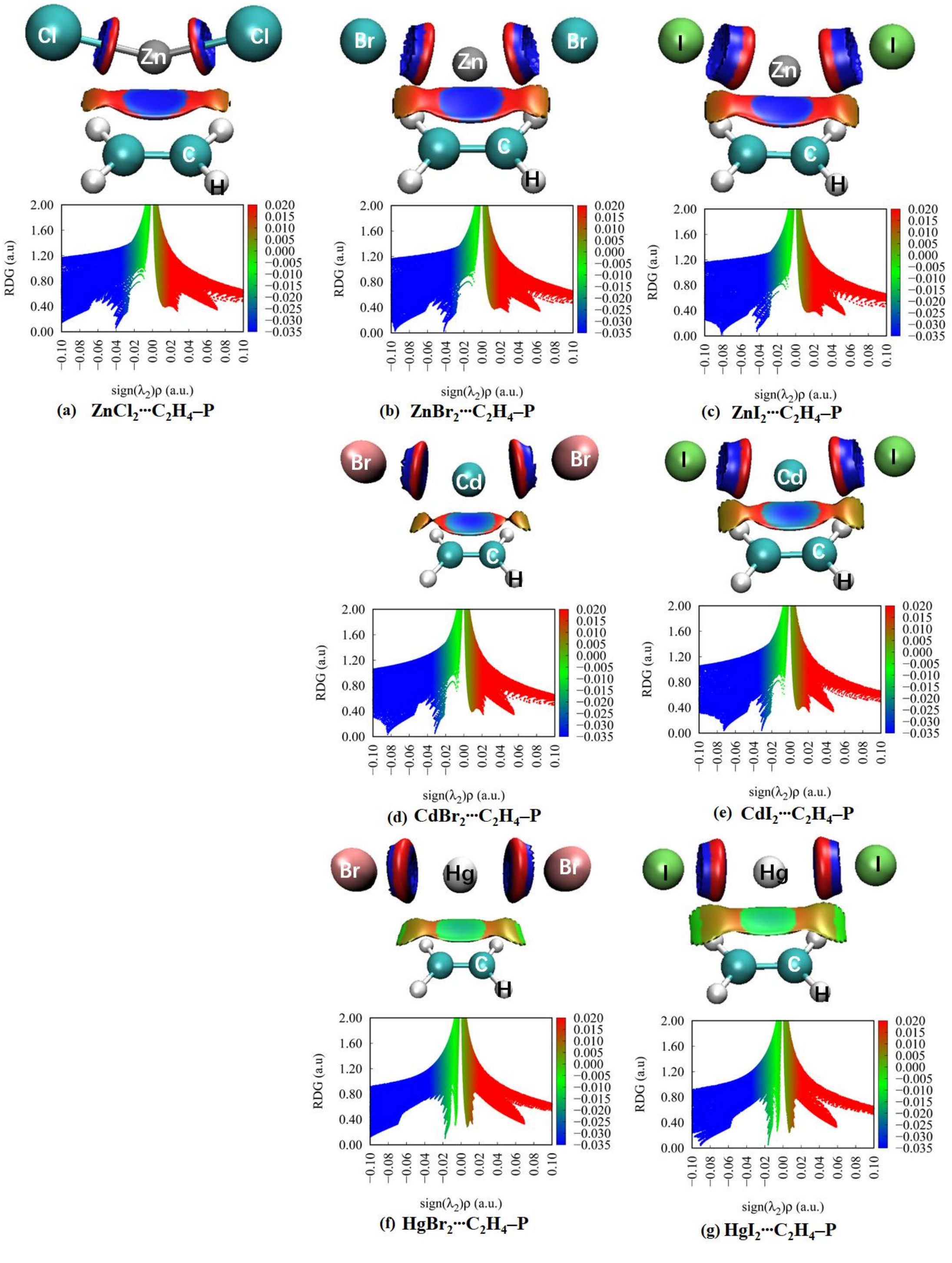


**Figure S4.** Plots of the reduced density gradient RDG *versus* the electron density multiplied by the sign of the second Hessian eigenvalue ($\mathrm{sign}(\lambda_2)\rho$) of $MX_2\cdots C_2H_4$–P. NCI maps of the corresponding binary complexes. Blue, green, orange, and red areas correspond to strong attractive, weak attractive, weak repulsive, and strong repulsive interactions, respectively.

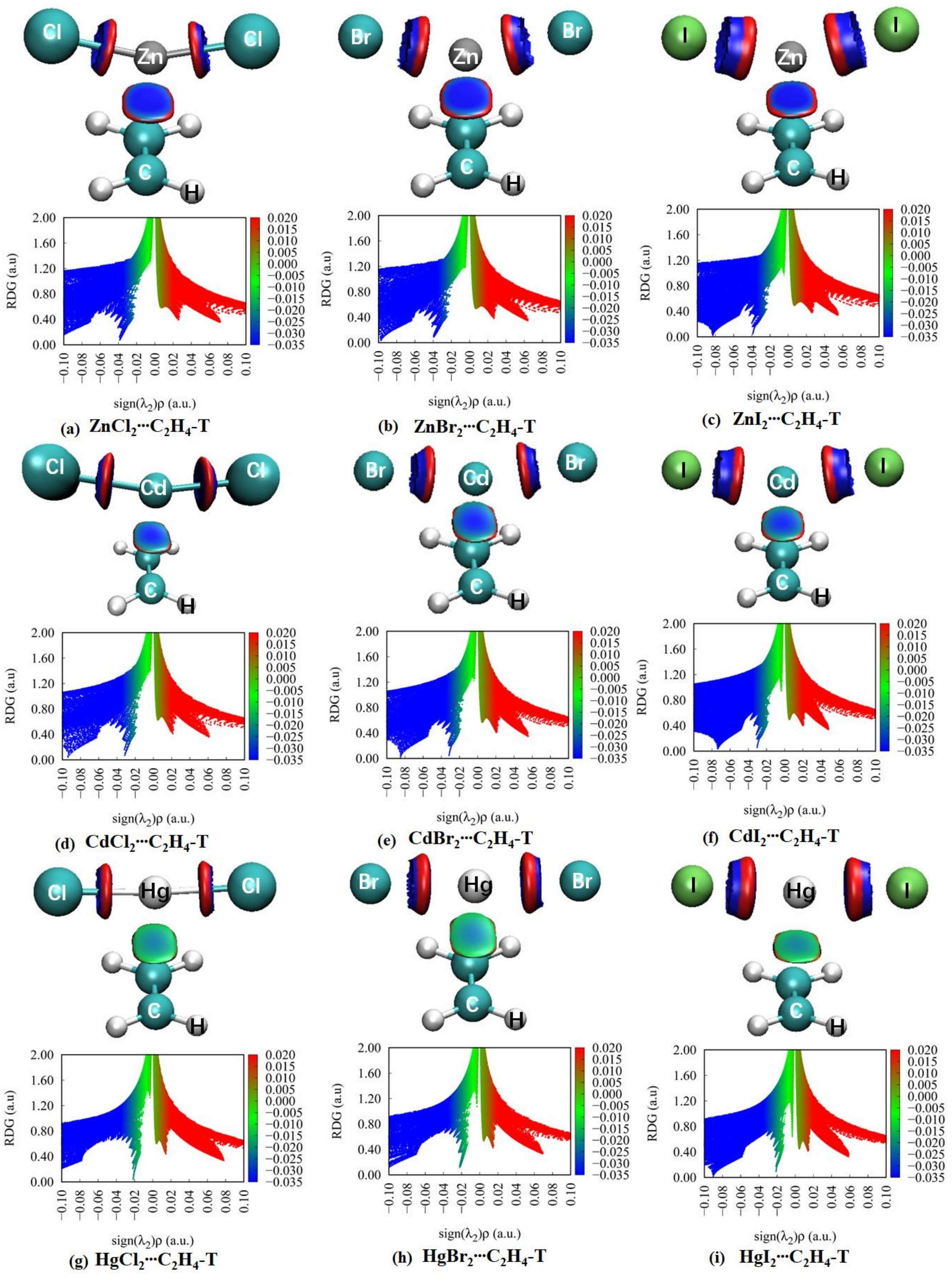


**Figure S5.** Plots of the reduced density gradient RDG *versus* the electron density multiplied by the sign of the second Hessian eigenvalue (sign($\lambda_2$)ρ) of $MX_2\cdots C_2H_4$–T. NCI maps of the corresponding binary complexes. Blue, green, orange, and red areas correspond to strong attractive, weak attractive, weak repulsive, and strong repulsive interactions, respectively.

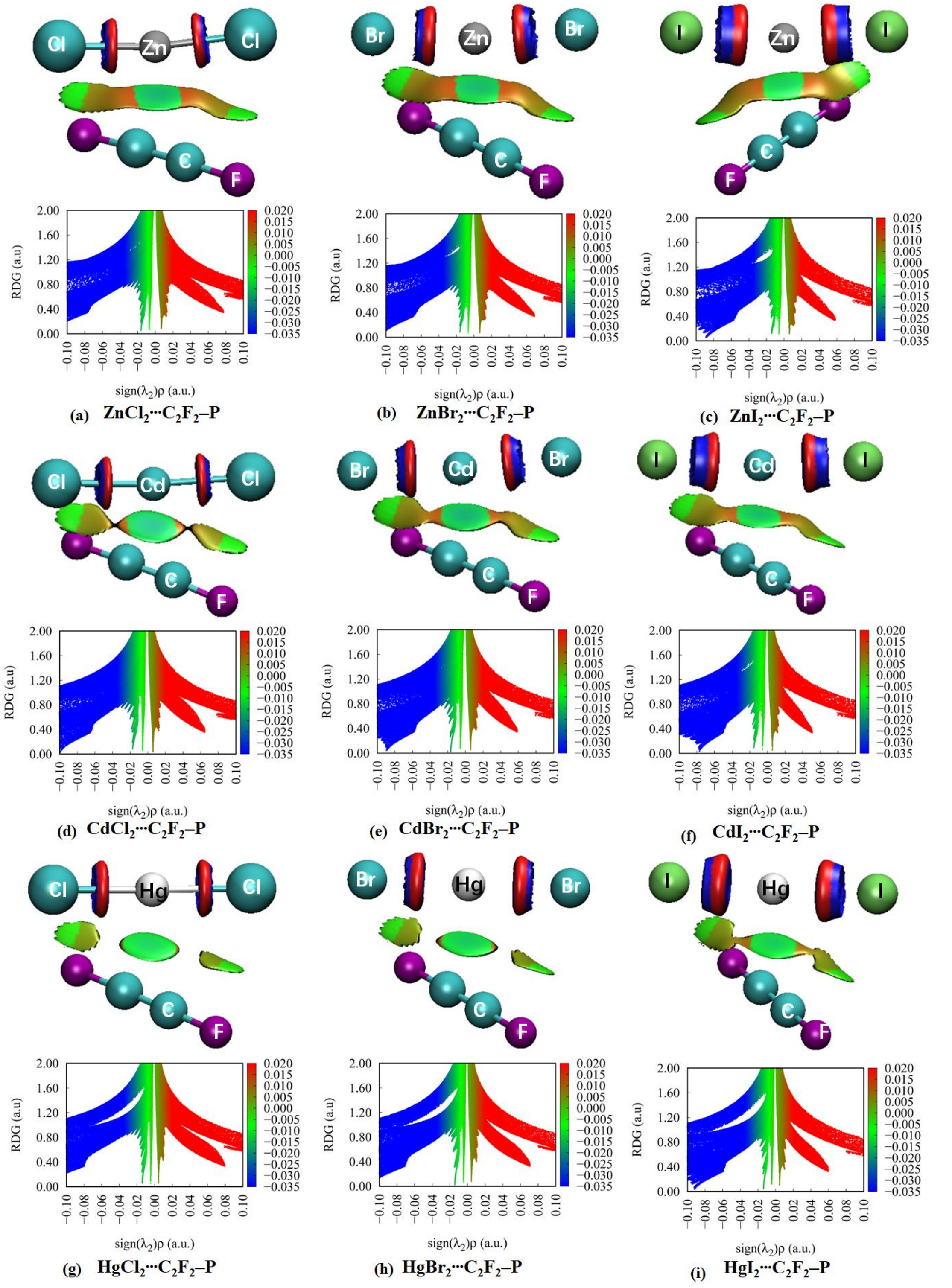


**Figure S6.** Plots of the reduced density gradient RDG *versus* the electron density multiplied by the sign of the second Hessian eigenvalue (sign($\lambda_2$)ρ) of $MX_2\cdots C_2F_2$–P. NCI maps of the corresponding binary complexes. Blue, green, orange, and red areas correspond to strong attractive, weak attractive, weak repulsive, and strong repulsive interactions, respectively.

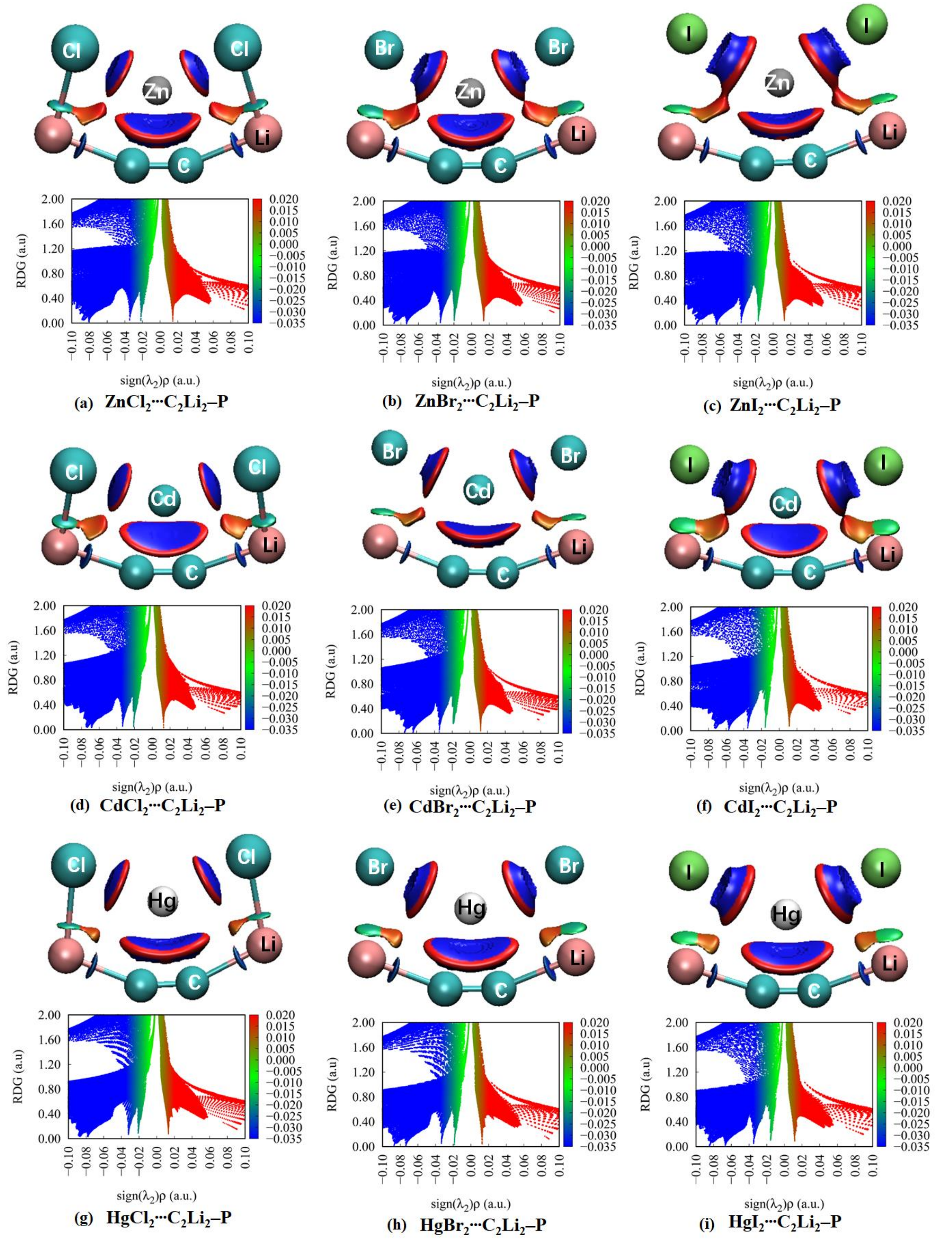


**Figure S7.** Plots of the reduced density gradient RDG *versus* the electron density multiplied by the sign of the second Hessian eigenvalue (sign($\lambda_2$)ρ) of $MX_2 \cdots C_2Li_2$–P. NCI maps of the corresponding binary complexes. Blue, green, orange, and red areas correspond to strong attractive, weak attractive, weak repulsive, and strong repulsive interactions, respectively.

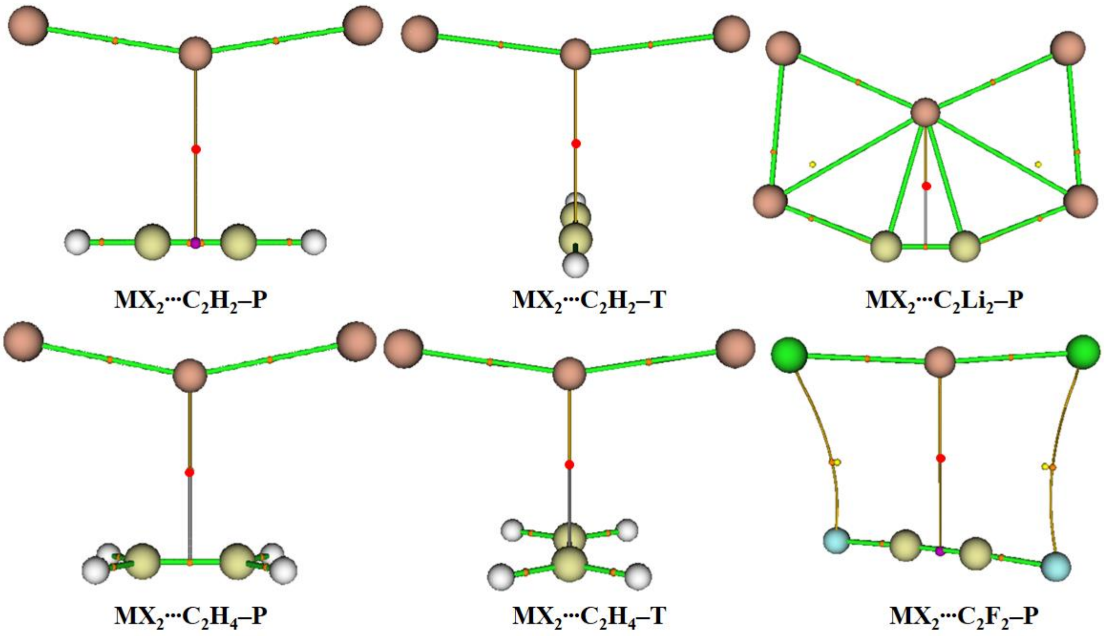


**Figure S8.** Molecular graphs of spodium–π bonded stable complexes at the intermolecular bond critical points.

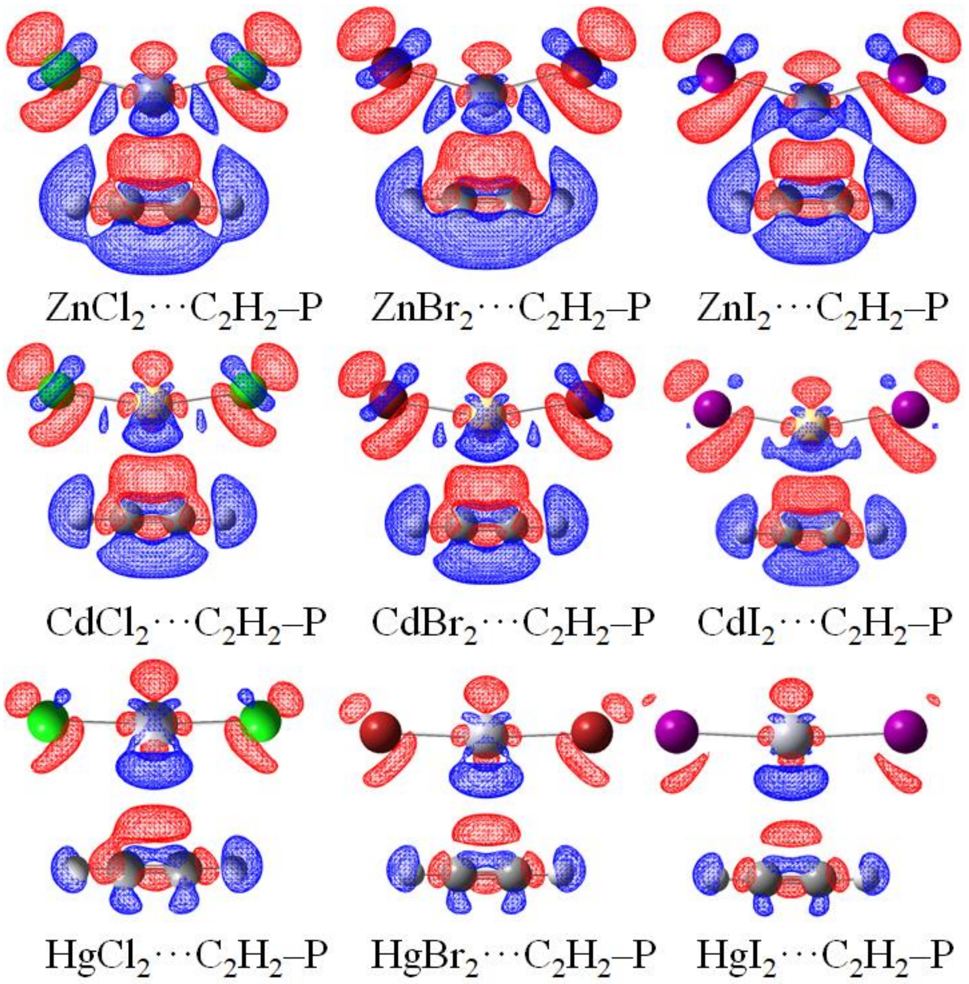


**Figure S9.** Electron density shifts of the $MX_2\cdots C_2H_2$–P complexes (iso = ± 0.0008). Red regions indicate increased electron density, while blue regions represent decreased electron density.

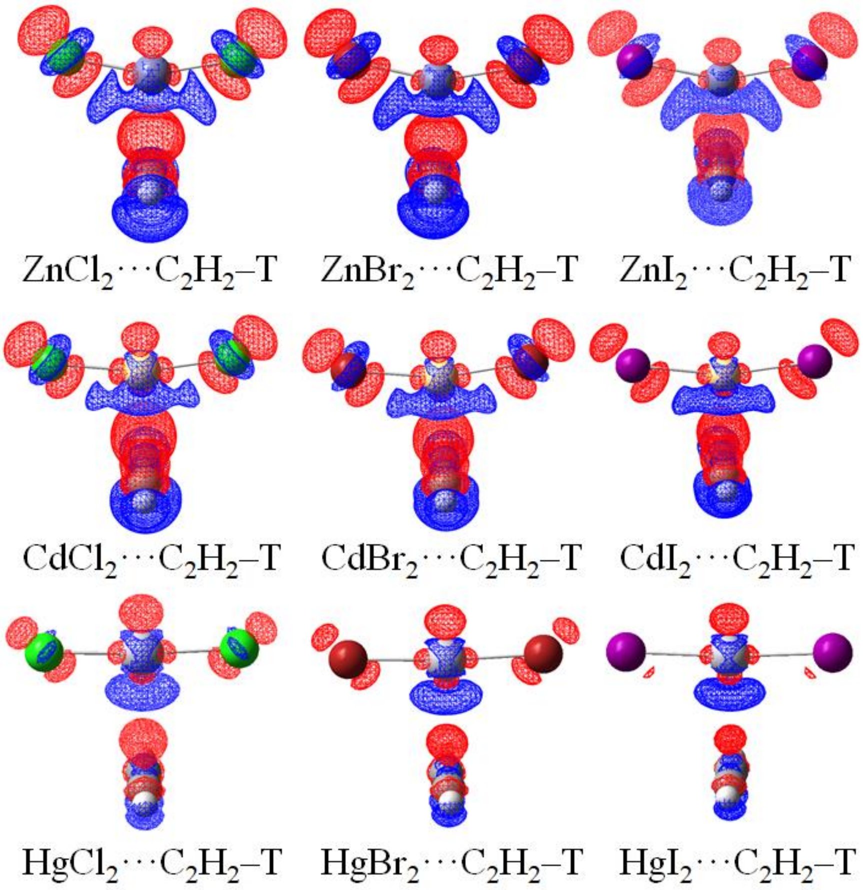


**Figure S10.** Electron density shifts of the $MX_2\cdots C_2H_2$–T complexes (iso = ± 0.0008). Red regions indicate increased electron density, while blue regions represent decreased electron density.

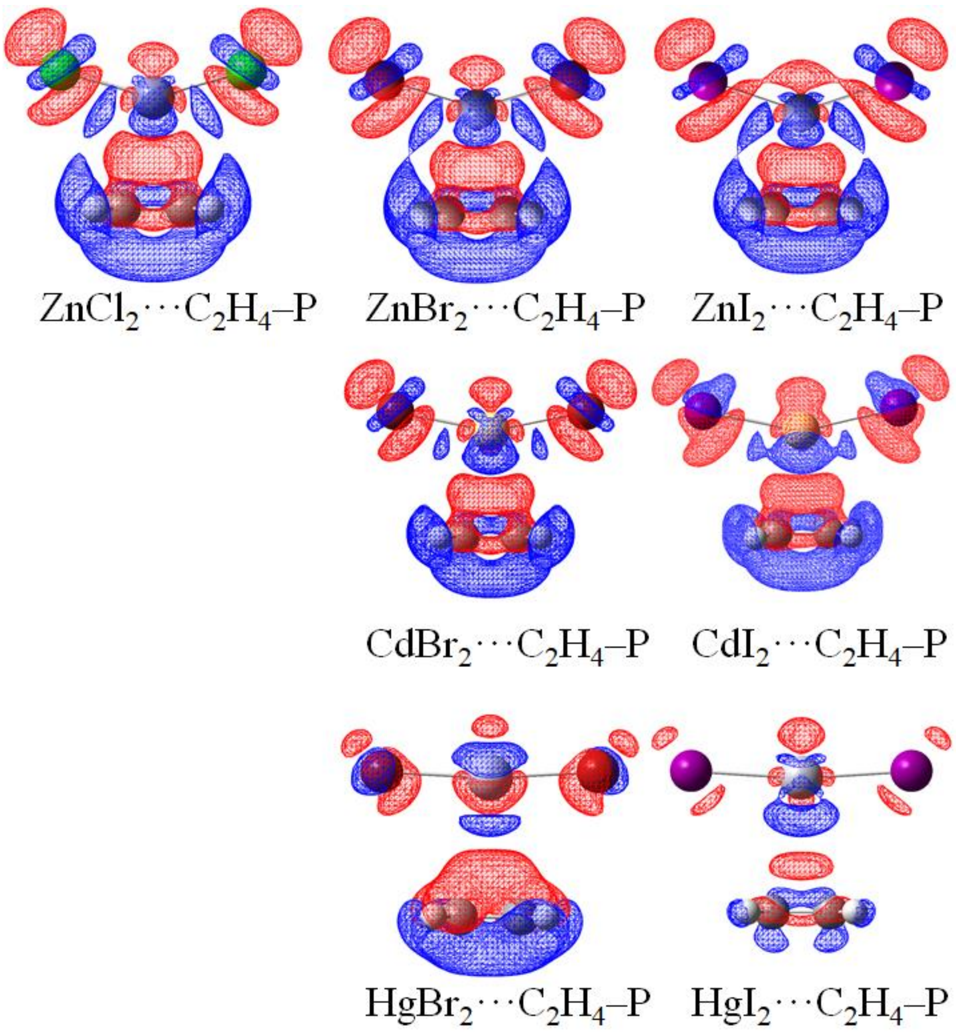


**Figure S11.** Electron density shifts of the $MX_2\cdots C_2H_4$–P complexes (iso = ± 0.0008). Red regions indicate increased electron density, while blue regions represent decreased electron density.

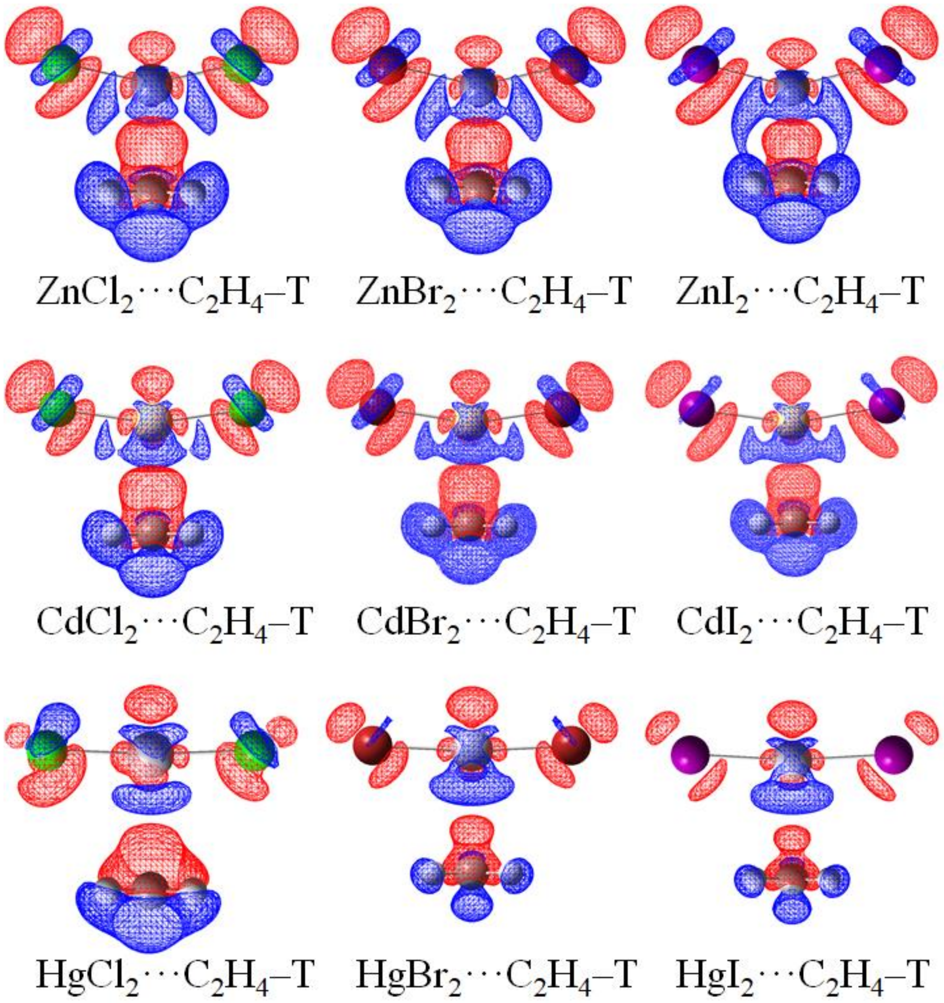


**Figure S12.** Electron density shifts of the $MX_2 \cdots C_2H_4$-T complexes (iso = ± 0.0008). Red regions indicate increased electron density, while blue regions represent decreased electron density.

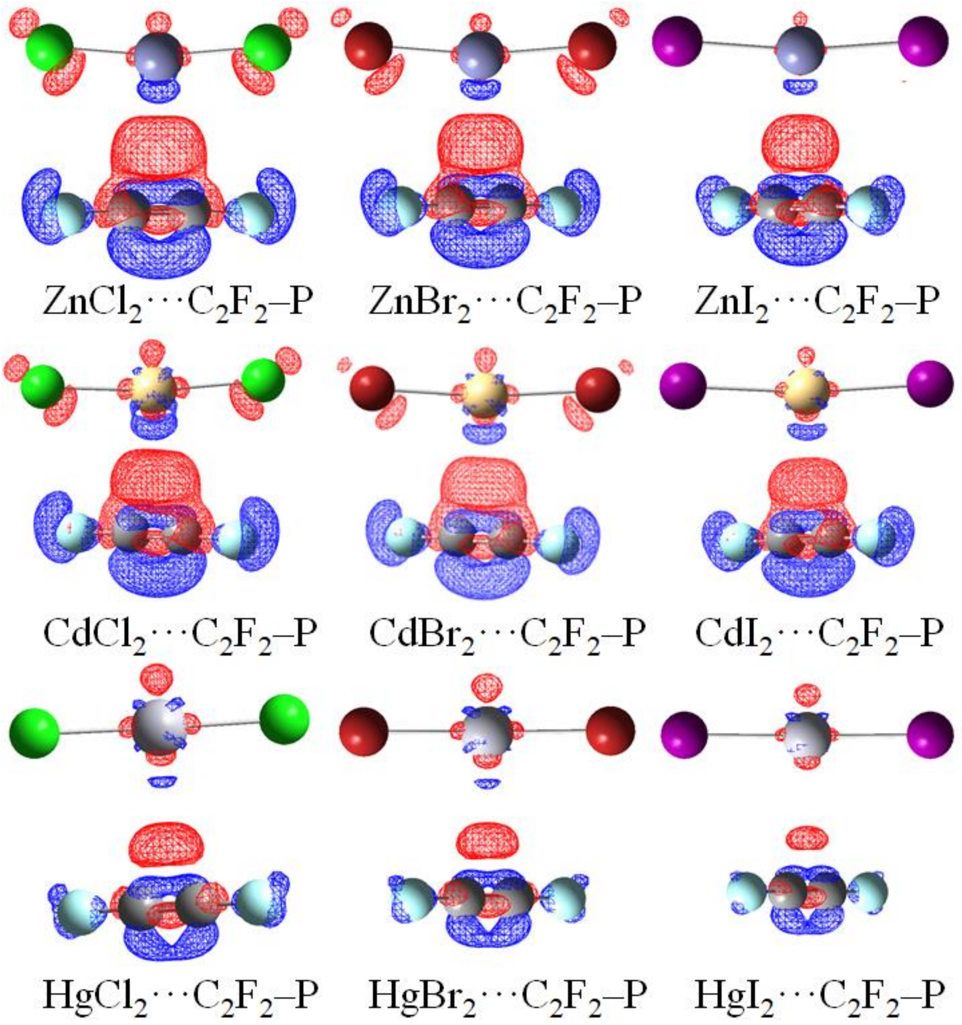


**Figure S13.** Electron density shifts of the $MX_2$···$C_2F_2$–P complexes (iso = ± 0.0008). Red regions indicate increased electron density, while blue regions represent decreased electron density.

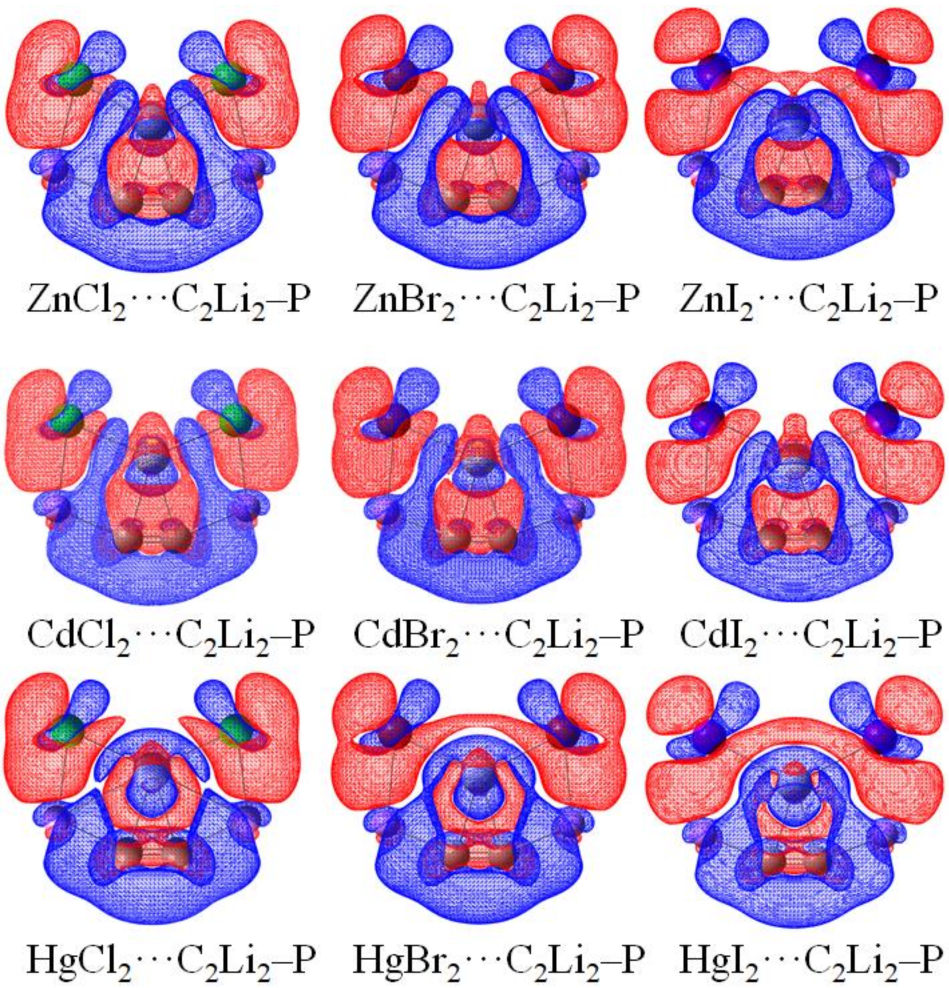


**Figure S14.** Electron density shifts of the $MX_2\cdots C_2Li_2$–P complexes (iso = ± 0.0008). Red regions indicate increased electron density, while blue regions represent decreased electron density.

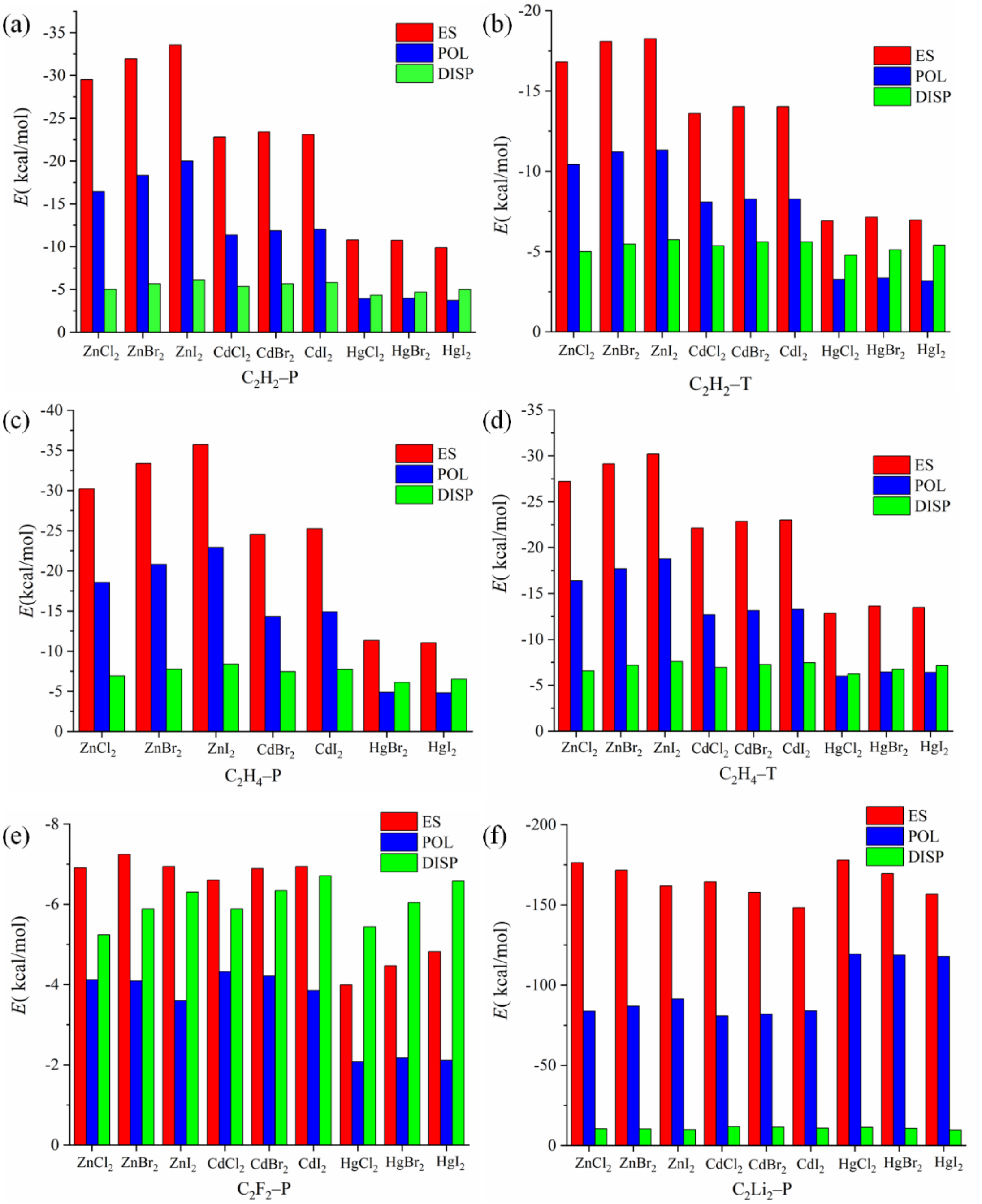


**Figure S15.** Comparison of electrostatic (ES), polarization (POL), and dispersion (DISP) energies in the binary complexes.
